# A Versatile Safeguard for Chimeric Antigen Receptor T-Cell Immunotherapies

**DOI:** 10.1038/s41598-018-27264-w

**Published:** 2018-06-12

**Authors:** Julien Valton, Valerie Guyot, Bijan Boldajipour, Cesar Sommer, Thomas Pertel, Alexandre Juillerat, Aymeric Duclert, Barbra Johnson Sasu, Philippe Duchateau, Laurent Poirot

**Affiliations:** 1 Cellectis Inc, 430E, 29th Street, NYC, NY 10016 USA; 2 Cellectis S.A., 8 rue de la Croix Jarry, 75013 Paris, France; 3 Pfizer Inc/Rinat, 230 E Grand Avenue, South San Francisco, CA 94114 USA

## Abstract

CAR T-cell therapies hold great promise for treating a range of malignancies but are however challenged by the complexity of their production and by the adverse events related to their activity. Here we report the development of the CubiCAR, a tri-functional CAR architecture that enables CAR T-cell detection, purification and on-demand depletion by the FDA-approved antibody Rituximab. This novel architecture has the potential to streamline the manufacturing of CAR T-cells, allow their tracking and improve their overall safety.

## Introduction

In less than a decade, adoptive CAR T-cell therapies have yielded unprecedented frequencies of complete remissions in hematological indications^1^. Despite this success, safety concerns have been raised regarding mild to life-threatening adverse effects related to CAR T-cells activity^2^. To address these potential concerns, different molecular safeguards were developed (Supplementary Table I)^3^. However, while these safeguards enable efficient on-demand depletion of engineered T-cells^4–11^, each of them display specific drawbacks including their size, potential immunogenicity^12^ and reliance on unapproved small molecules as activating agent^4,5^ (Supplementary Table I). In addition, all of them are presented at the cell surface separated from the CAR, an architecture that could potentially lead to unbalanced CAR/safeguard ratio and allow safeguard-free CAR T-cell populations to emerge. We thus sought to evaluate an alternative strategy by integrating a compact safeguard within the CAR to generate an all-in-one architecture. Here we report the development of a CAR architecture that in addition to allowing universal detection and purification of the resulting CAR T-cells, enables their fast and efficient eradication by the FDA-approved antibody Rituximab (RTX).

**Figure 1:**
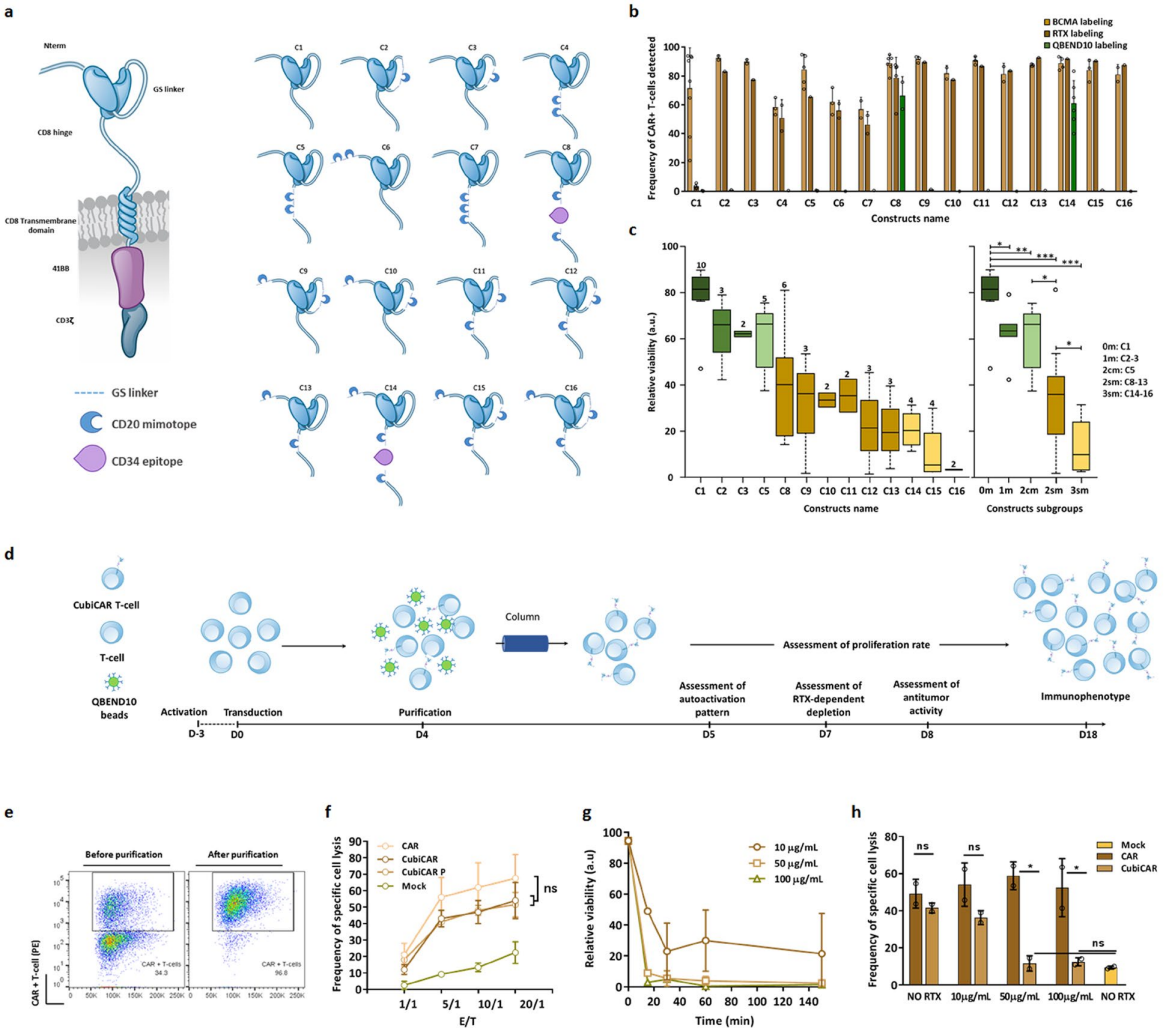
*In vitro* screening, identification and characterization of the CubiCAR architecture. (**a**, left panel) Scheme of the 2^nd^ generation CAR construct used in this study. This construct includes an anti-BCMA ScFV, a CD8 hinge and transmembrane domain, a 4-1BB costimulatory domain and a CD3 activation domain (**a**, right panel) Scheme and names of the different engineered extracellular constructs tested. The location of CD20 mimotopes and CD34 epitope are indicated. (**b**) Flow cytometric detection of CAR constructs transiently expressed at the surface of primary T-cells using either the soluble BCMA protein, RTX or QBEND10 as surface markers. The error bars in represent the standard deviation on experimental values computed out of ≥2 biological replicates performed with ≥2 different donors (**c**) Box plot illustrating the median of efficiency of RTX-dependent depletion of primary T-cells transiently expressing CAR constructs. Viability of primary T-cells incubated for 150 min in the presence of 100 µg/mL RTX and complement was determined by flow cytometry and normalized to untreated control (relative viability, see Methods). Relative viability is indicated for each constructs (left panel) or for construct subgroups including those containing 2 consecutive CD20 mimotopes (2 cm) and 2 to 3 separated CD20 mimotopes (2 sm, 3 sm respectively, right panel). The number of independent biological replicates performed is indicated at the top of each box plot. The significance of the differences between subgroups was assessed using a non parametric Mann-Whitney U test (ns, non significant, *p < 0.05, **p < 0.01, ***p < 0.001). (**d**) Schema of the workflow used to characterize primary T-cells steadily expressing the CubiCAR (C14) construct. (**e**) Flow cytometry analysis of CubiCAR T-cells before and after QBEND10 coated beads purification using BCMA soluble protein as surface marker. (**f**) Specific cell lysis activity of unpurified and purified CubiCAR T-cells toward BCMA^+^ and BCMA- tumor cell lines determined at different E/T ratio. (**g**) Kinetic of CubiCAR T-cells depletion by complement and increasing amounts of RTX (10–100 µg/mL). (**h**) Effect of RTX on the specific cell lysis activities of CAR or purified CubiCAR T-cells. Activities were determined after a 30 min long incubation of cells with complement and increasing amounts of RTX. The Error bars in (**f**), (**g**) and (**h**) represent the standard deviation on experimental values (technical triplicate) computed out of 2 biological replicates performed with 2 different donors. The significance of the differences between subgroups in (**f**) and (**h**) was assessed using a one-way ANOVA test (ns, non significant, *p < 0.05, **p < 0.01, ***p < 0.001). Reproduced with permission from Cellectis group.

To identify an optimal safeguard CAR architecture, we assembled 14 different constructs containing 1, 2 or 3 CD20 mimotopes that were reported to be non-immunogenic and specific for RTX binding^9^ (Supplementary Table II). These mimotopes were engrafted at different positions of the extracellular portion of a 2^nd^ generation CAR construct^13^ designed to target B cell maturation antigen- (BCMA), an antigen reported to be relevant to treat multiple myeloma^14^ (Fig. 1a, Supplementary Table II). Two additional constructs containing a human CD34 epitope reported earlier to allow for efficient cell enrichment^9^, were also assembled. For throughput considerations, all constructs were first transfected in primary T-cells as mRNA and screened one day post transfection for their ability to be expressed on the surface of T-cells, to allow depletion by RTX and to stimulate anti-tumor activity.

All constructs (C1-C16) were expressed and able to bind to soluble BCMA protein (Fig. 1b and Supplementary Fig. 1). Percentage of CAR+ T-cells ranged from 70% to more than 90% for all but three constructs, C4, C6 and C7, which were excluded from further investigation for this reason. CAR constructs could also be labeled by RTX (C2-C16) or by the anti-CD34 antibody QBEND10 (C8 and C14) indicating that the integrated CD20 mimotopes and CD34 epitope were properly folded and able to bind efficiently to both antibodies (Fig. 1a and Supplementary Fig. 1b). As expected, such binding was not observed in the unmodified C1 CAR architecture lacking epitopes. CAR detection using soluble BCMA protein, RTX or QBEN10 was similar across different constructs, demonstrating that RTX and QBEND10 could be used as reliable detection probes of CAR surface expression.

The ability of CAR constructs to promote T-cell depletion was then evaluated using RTX-mediated complement dependent cytotoxicity (CDC) assays. Primary T-cells transiently expressing CAR constructs were incubated in the presence of RTX and complement and analyzed by flow cytometry. Determination of CAR+ T-cells viability enabled us to observe a wide range of depletion efficiencies (Fig. 1c, left panel and Supplementary Fig. 2). Depletion efficiencies, defined here as the decrease of viability due to treatment, positively correlated to the number of CD20 mimotopes present in the CAR construct and varied from 30% to almost 100% (Fig. 1c, left panel). Interestingly, positioning epitopes in two separate locations within the molecule achieved significantly better depletion than having two consecutive epitopes within the same location (contruct subgroups 2 cm and 2sm respectively, Fig. 1c, right panel, Table II). We speculate that spatial separation of CD20 mimotopes may improve binding efficiency by reducing the steric hindrance generated between multiple rituximab molecules.

In addition to being detectable and depletable by RTX, primary T-cells transiently expressing CAR constructs showed cytolytic activity toward antigen-expressing tumor cells. Indeed, by analyzing the remaining viability of BCMA-expressing cells (H929) incubated with CAR T-cells, we found that all constructs tested were able to promote target cell killing to a similar magnitude as the unmodified C1 CAR counterpart (Supplementary Fig. 3).

Overall, our screening results showed that the majority of CAR constructs were expressed at the surface of T-cells, endowed them with cytolytic activity toward BCMA-expressing tumor cell lines and enabled their depletion by RTX. Depletion by RTX was optimal for CAR architectures containing 3 separated CD20 mimotopes. Among these architectures, C14 was chosen for further investigation because it bore an integrated CD34 epitope that could enable cell sorting and enrichement of CAR positive T-Cells. This architecture will be referred to as CubiCAR (3 functions integrated in the CAR: depletion, detection and purification) in the following sections and for the sake of simplicity, its unmodified counterpart will be called CAR.

To investigate the functional properties of the CubiCAR architecture and evaluate its potential for downstream clinical applications, primary T-cells were transduced with lentiviral particles encoding CubiCAR and characterized *in vitro* for a variety of biological aspects (Fig. 1d). We first assessed the ability of CubiCAR T-cells to proliferate after being purified. Transduced primary T-cells (MOI 2, 3 days post CD3/CD28 activation) were cultured for 4 days before being purified using GMP-compatible CD34-specific magnetic beads. CubiCAR T-cells could be purified to >96% purity with an average rate of recovery of 60%, an efficiency comparable to those obtained with other tagged-CAR architectures (Fig. 1e and Supplementary Fig. 4)^15,16^. Comparison of growth rates obtained for CAR, purified (CubiCAR P) and non-purified (CubiCAR) T-cells showed a similar expansion level (Supplementary Fig. 5) indicating that the proliferative potential of CAR T-cells was neither affected by the presence of CubiCAR architecture nor by the purification process. Consistent with this, CubiCAR engagement by CD34-specific beads during the purification process did not lead to T-cell activation as no CD69 upregulation was observed one day post purification (CD69+ upregulation in CubiCAR P and CubiCAR, Supplementary Fig. 6). Finally, comparison of T-cell and CAR T-cell immunophenotypes obtained after 14 days of culture showed similar CD8 memory and effector subsets percentages with a majority of T central memory (Tcm, Supplementary Fig. 7). This result confirmed that CubiCAR expression at the surface of primary T-cells had a negligible impact on their differenciation level and ability to proliferate.

We then investigated the cytolytic activity and specificity of CubiCAR T-cells toward BCMA-expressing cell lines. To do so, CubiCAR T-cells were incubated for 5 hours in the presence of a stoichiometric mixture of BMCA^+^ and BCMA- labeled target cell lines, at variable effector/target (E/T) ratio. Consistent with the first activity screening described earlier, CubiCAR P, CubiCAR and CAR T-cells both displayed cytolytic activity (Fig. 1f). A similar conclusion was reached when the antitumor activity of CubiCAR P and CAR T-cells was challenged by daily addition of fresh tumor cells over a 12 days period using a long term serial killing assay (Supplementary Fig. 8). In addition, CubiCAR and CAR T-cells showed negligible activity toward BCMA- cells (data not shown) and similar BCMA binding profile (Supplementary Fig. 9) indicating that CubiCAR modifications do not impact CAR target binding capacity and specificity.

We further assessed the sensitivity of CubiCAR T-cells to RTX treatment and characterized the kinetics of depletion in CDC assays. Our results first showed that CubiCAR P T-cells were fully depleted by the RTX treatment (Supplementary Fig. 10), an efficiency similar to earlier results obtained on safeguard systems associated with CAR^4,5,7,9^ or TCR^17^. Depletion kinetics of CubiCAR T-cells were dependent on RTX concentration and occurred with a half-life of approximately 10 min in the presence of 50 µg/mL RTX, a concentration about 10-fold lower than the RTX Cmax reported in patients (Fig. 1g)^18^. Consistent with this, RTX profoundly and rapidly inhibited CubiCAR T-cells as a 30 min-long incubation with 50 µg/mL RTX and complement was sufficient to abrogate their overall anti-tumor activity (Fig. 1h). Thus, our results showed that CubiCAR T-cells could be efficiently purified, retained their capacity to proliferate and specifically kill tumor cells, and have the ability to be rapidly depleted by a clinically relevant dose of RTX.

Because of its potential to improve the safety of CAR T-cell immunotherapies, we tested the general applicability of the CubiCAR architecture with other CARs. To do so, we replaced the anti-BCMA scFv model with 4 unrelated scFvs^19–22^ shown to efficiently eradicate different malignancies when embedded in a CAR^13,23–25^. The resulting CubiCAR constructs were detected at the surface of transduced T-cells using RTX and QBEND10 dual labeling or their respective soluble protein target (Supplementary Fig. 11) and enabled their purification without promoting significant T-cell activation (Supplementary Figs 12 and 13). CubiCAR T-cells displayed similar proliferation rates compared to their CAR counterparts (Supplementary Fig. 14) and were efficiently eradicated by RTX (Supplementary Fig. 15). Furthermore, when transiently or constitutively expressed at the surface of T-cells, all CubiCAR constructs promoted specific anti-tumor activity in the range of those obtained with the respective CAR counterparts (Supplementary Fig. 16). Thus, the CubiCAR architecture could be considered as a transportable versatile safeguard. In that sense, it could be readily implemented as a scaffold architecture in the conventional scFvs screening that is usually required to select optimal CAR constructs for a given tumor antigen.

**Figure 2:**
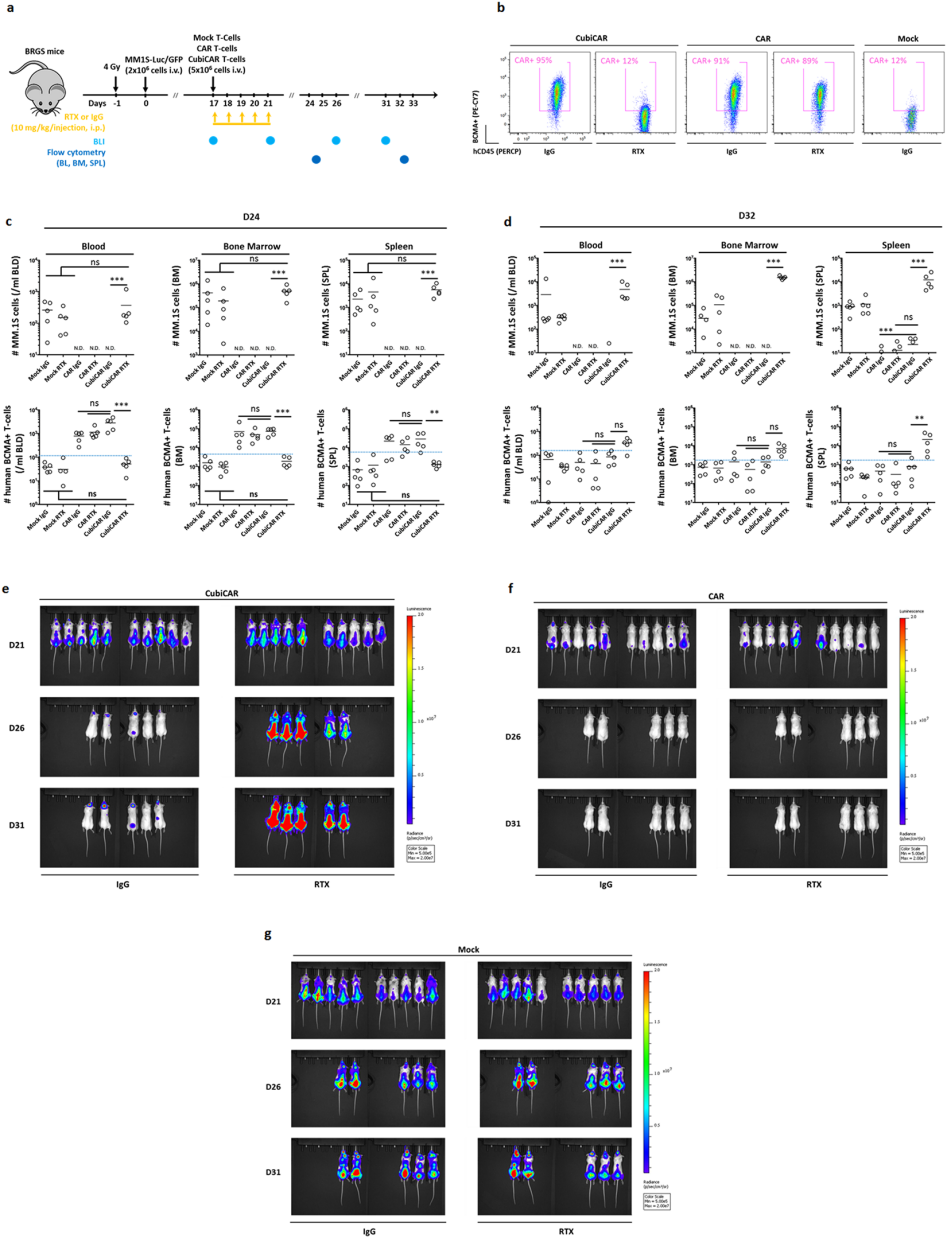
CubiCAR T-cells display anti-tumor activity and are specifically depleted by RTX *in vivo*. (**a**) Experimental workflow used to assess the anti-tumor activity of purified CubiCAR T-cells and the extent of their depletion by RTX in BRGS mice. Bioluminescence (BLI) and flow cytometry analysis of blood, bone marrow and spleen (BLD, BM and SPL respectively) are indicated by light and dark blue dots respectively. Mice were imaged at D17 for randomization and at D21, D26 and D31 to monitor tumor expansion. 5 mice/group were sacrificed at D24 and D32 for flow cytometry analysis. (**b**) and (**c**) results of flow cytometry analysis obtained at D24 and D32 respectively and expressed as number of human MM.1 S or BCMA+ T-cells (defined as T-cells, positively labeled by soluble biotinylated-BCMA protein and fluorescent streptavidin). MM.1S-Luc-GFP cells were detected as CD38+ and GFP+ among mCD45−/hCD45- cells (supplementary Fig. 16a). CAR and CubiCAR T-cells were detected by the soluble biotinylated BCMA protein staining (BCMA+) among the hCD45+/CD3+ cells. The threshold of BCMA + T-cells detection, set by the highest signal obtained from Mock transduced T-cells, is indicated by a dashed blue line. Mock transduced T-cells (Mock) were detected among hCD45+ cells as CD3+/BCMA− cells (supplementary Fig. 16a). (**d**) Representative flow cytometry results obtained in at D24, from the BM of mice infused by CubiCAR, CAR and Mock transduced T-cells in the presence of IgG or RTX. (**e**), (**f**) and (**g**) bioluminescence analysis of mice respectively infused by CubiCAR, CAR or mock transduced T-cells at D21, D26 and D31 in the presence or in the absence of RTX. All pictures illustrate the radiance (p/s/cm2/sr) of luminescence that was computed with the same dynamic range for each mouse. N.D, non detectable. Data were log-transformed before doing an ANOVA followed by a multiple comparison Tukey HSD test (ns, non-significant, *p < 0.05, **p < 0.01, ***p < 0.001). Reproduced from the Cellectis group except for the mouse model scheme that is not subject to copyright and was obtained from Pixabay web site.

Having demonstrated the versatility and broad applicability of CubiCAR T-cells *in vitro*, we investigated their anti-tumor activity and depletability *in vivo*. For that purpose, we used luciferase-GFP expressing human MM.1 S cell as a BCMA^+^ tumor target and an immunodeficient BRGS mouse model. This mouse model is deficient of T, B and NK cells but have a functional complement system^26,27^, allowing us to assess the potency of RTX via CDC activity^26,27^. Two complementary readouts, bioluminescence live imaging of mice and flow cytometry analysis of their blood, bone marrow and spleen were used to monitor tumor expansion as well as proliferation and RTX-mediated depletion of mock transduced, CAR, and CubiCAR P T-cells (Fig. 2a).

In the absence of RTX, injection of anti-BCMA CAR or CubiCAR T-cells was sufficient to eradicate MM.1 S in blood, bone marrow and spleen. This was demonstrated by flow cytometry analysis, showing undetectable or negligible levels of MM.1 S cells at the two time points. (Fig. 2b,c, upper panels and Supplementary Fig. 17). Efficient anti-tumor activity of CAR and CubiCAR T-cells was confirmed by the reduction of MM.1 S bioluminescence signal over time, although residual signal could be still detectable in brains of mice treated with CubiCAR T-cells (Fig. 2e,f). In contrast, injection of RTX in mice treated with CubiCAR T-cells resulted in an unchecked proliferation of MM.1 S cells as demonstrated by a high bioluminescence signal (Fig. 2e) and a high number of MM.1 S cells in the three tissues (Fig. 2b,c, upper panels). This remarkable effect was correlated with a significant decrease of CubiCAR T-cell number that dropped below the detection threshold set by mock transduced control T-cells at D24 (Fig. 2b, lower panel and Fig. 2d). In stark contrast, MM.1 S cells were still efficiently eliminated by CAR T-cells that were unaffected by RTX treatment, indicating that RTX depletion potency was specific to CubiCAR T-cells (Fig. 2d, Fig. f and Fig. 2b,c, lower panels compare CAR IgG and CAR RTX conditions). Interestingly, CubiCAR T-cells could be detected at the end of the study, despite having been largely depleted by earlier RTX treatment (Fig. 2b,c lower panels, compare CubiCAR RTX conditions). This may be due to the continued presence of disease in these mice, leading to strong re-stimulation and proliferation of the few remaining CubiCAR T-cells at this later time point (Fig. 2c, upper panel, see CubiCAR RTX condition). This suggests that depending on the dose, number and timing of RTX injections, the activity of CubiCAR T-cells may be tunable as demonstrated for another safeguard system^28^. Taken together and in agreement with our *in vitro* dataset, our results showed that CubiCAR T-cells display efficient anti-tumor activity and can be readily and specifically depleted by RTX *in vivo*.

Here, we report the development of an alternative safeguard for chimeric antigen receptor T-cell therapies. We have incorporated a safeguard component into a conventional CAR architecture that enables tight control over CAR T-cell activity and has the potential to improve their safety profile in clinical settings. The CubiCAR architecture comprises an integrated, compact safeguard allowing fast and efficient depletion of CAR T-cells by the FDA-approved antibody RTX. CubiCAR T-cells retain their proliferative potential and showed efficient anti-tumor activity. Besides its anti-tumor and safety features, the CubiCAR architecture has the advantage of enabling purification and detection of CAR T-cells by commercially available and GMP-compatible antibodies. These two additional integrated features will help controlling and standardizing the amount of active biological material per clinical batch, particularly for autologous therapies, which have been shown to be susceptible to patient-to-patient variability^29^. In addition, the CubiCAR architecture is compatible with multiple scFvs designed against different targets, thus bearing the potential to improve the safety of CAR T-cell immunotherapies for a broad range of patients.

## Methods

### Materials

Cell culture reagents, X-vivo-15 was obtained for Lonza (cat#BE04-418Q), IL-2 from Miltenyi Biotech (cat#130-097-748), human serum AB from Seralab (cat#GEM-100–318), human T activator CD3/CD28 from Life Technology (cat#11132D), CD34 MicroBead kit and MACS® LD-column from Miltenyi Biotech (cat#130-046-702 and cat#130-042-901 respectively), QBEND10-APC from R&D Systems (cat#FAB7227A), Rituximab from internal sources, Baby rabbit complement (cat#C12CA) from AbD Serotec/BIO-RAD, eFluor 780 from eBioscience (cat# 65-0865-14) and retronectine form Takara (cat#T100B). Cryopreserve human PBMC, acquired from Allcells (cat#PB006F) were used in accordance with all applicable Pfizer policies, including IRB/IEC approval.

### Transient expression of CAR constructs in primary T-cells

Primary T-cells isolated from PBMC (ALLCELLS) were activated and transfected according to previously described methods^13^. Four days after their activation by Dynabeads human T activator CD3/CD28, 5 × 10^6^ T-cells were transfected with 30 µg of mRNA encoding CAR constructs. Transfection was performed using Pulse Agile technology, by applying two 0.1 mS pulses at 3,000 V/cm followed by four 0.2 mS pulses at 325 V/cm in 0.4 cm gap cuvettes and a final volume of 200 µl of Cytoporation buffer T (BTX Harvard Apparatus, Holliston, Massachusetts). Cells were then immediately diluted in X-Vivo-15 media supplemented by 20 ng/ml IL-2 (final concentration) and 5% human serum AB. Transfected T-Cells were eventually diluted at 1 × 10^6^/ml and kept in culture at 37 °C in the presence of 5% CO_2_ and 20 ng/ml IL-2 (final concentration) and 5% human AB serum for further characterization.

### Lentiviral particles generation and transduction of primary T-cells

Lentiviral particles were generated in 293 H cells, concentrated by ultracentrifugation and titrated in Jurkat cells using CAR expression as transduction read out as previously described^30^. 1 × 10^6^ primary T-Cells (resuspended in 600 µL of X-vivo-15 media) were plated 3 days post activation on a 12 well plate pre-coated by 30 µg/mL retronectin in the presence of lentiviral particles to get an MOI of 2. After 2 H incubation at 37 °C, 600 µl of complete media 2 × (X-vivo-15, 10% AB serum and 40 ng/ml IL-2) was added to the cellular suspension. After overnight incubation, cells were washed, resuspended in a complete media at 1 × 10^6^ cells/mL and passaged every 2 or 3 days.

### Isolation of CubiCAR T-cells using magnetic separation

CubiCAR T-cells were purified 4 days post transduction using the CD34 MicroBead kit and MACS-LD column from Miltenyi Biotech according to the manufacturer protocol. Briefly, 5 to 10 × 10^7^ T-cells were incubated with 100 µL of FC-blocking reagent and 125 µL QBEND10 coated magnetic beads for 30 min in a final volume of 500 µL CliniMACS Buffer. Cellular suspension was then loaded onto a MACS® LD-Column attached to a conventional Myltenyi magnet and washed three times with 1 ml CliniMACS Buffer. Column was eventually removed from the magnet and CubiCAR T-cells were eluted by 3 mL of the same buffer. One round of purification was sufficient to obtain a homogeneous population of CubiCAR T-cells (purity > 96%) with a frequency CAR+ T-cells recovery of 60%. The frequency of CAR+ T-cells recovery was calculated according to the equation below:

Frequency CAR+ T-cells recovery = (Amount of purified CAR+ T-cells × 100)/Amount of CAR+ T-cells loaded onto the column.

### Flow-based cytotoxicity assay

The cytolytic activity and specificity of transduced CAR T-cell was assessed according to the previously described protocol^13^. This assay consisted of labeling 1 × 10^4^ tumor target positive cells and 1 × 10^4^ tumor target negative cells (L363 and Molm13 respectively for testing anti-BCMA CAR) with 1 µM CellTrace^TM^ CFSE and 1 µM CellTrace^TM^ violet respectively (Life Technology) and co-incubating them with 1 × 10^4^, 5 × 10^4^, 1 × 10^5^, and 2 × 10^5^ effector CAR T-cells (E/T ratio of 1/1, 5/1, 10/1 and 20/1) in a final volume of 100 µl X-Vivo-15 media, for 5 hours at 37 °C. Cells were then recovered and labeled with eFluor780 viability marker before being fixed by 4% PFA. Fixed cells were then analysed by flow cytometry to determine their viability. The frequency of specific cell lysis was calculated using the formula described below:

$${\rm{Frequency}}\,{\rm{of}}\,{\rm{specific}}\,{\rm{cell}}\,{\rm{lysis}}=({\rm{Via}}\,{\rm{L363}}+{\rm{T}}/{\rm{Via}}\,{\rm{Molm13}}+{\rm{T}})/({\rm{Via}}\,{\rm{L363}}/{\rm{Via}}\,{\rm{Molm13}})$$

where Via L363 + T and Via Molm13 + T correspond respectively to the % of viable L363 and Molm13 cells obtained after 5 hours in the presence of CAR T-cells and where Via L363 and Via Molm13 correspond respectively to the % of L363 and Molm13 cells obtained after 5 hours in the absence of CAR T-cells. Specific cell lysis comparisons between CAR and CubiCAR T-cells were performed with the same percentage of CAR positive T-cells. Percentage of CAR positive T-cells was adjusted by diluting CAR positive T-cells in Mock-transduced T-cells.

The flow based assay used for activity screenings of T-cells transiently expressing CAR constructs was performed according to the same conditions but only in the presence of H929 cells (BCMA- tumor cells) and at a E/T ratio of 1/10. Viability of H929 was determined for each construct except for C4, C6 and C7 that were excluded from this study because of their low CAR expression (nd, not determined). Of note, because percentage of CAR positive T-cells obtained one day post transfection was similar for all constructs tested, anti-tumor activities could be thus directly compared without adjustment.

### Long term serial killing assay

To assess the antitumor activity of transduced CAR T-cells over several round of tumor challenge CAR CubiCAR T-cells were added to a suspension of L363-Luc-GFP tumor cells (5 × 10^5^ total tumor cells, 2 mL final volume) at variable E/T ratio (E/T = 0.25; 0.125; 0.062) in Xvivo-15 media supplemented by 5% AB. The mixture was incubated 24 hours before measuring the luminescence signal of the mixture. Cells were then spun down and the media supernatant was discarded and substituted with 2 mL of fresh Xvivo 5% AB containing 5 × 10^5^ L363-Luc-GFP cells and the resulting cell mixture was incubated for 24 hours. This protocol was repeated for 12 days. Luminescence signals of L363-Luc cells incubated with variable amount of CubiCAR and CAR T-cells were determined every day and plotted as a function of time.

### Rituximab-dependent depletion assay

To assess the effect of RTX and complement on CAR T-cells viability, 2 × 10^5^ CAR T-cells were incubated alone or in the presence of 10–100 µg/mL RTX and complement (diluted 4 times from a 2 ml stock solution of baby rabbit complement, AbD serotec BIO-RAD, cat#C12CA) in a final volume of 400 µL. After variable time lengths (30–150 min), 200 µL of cellular suspension was recovered, washed with PBS, labeled by EFluor780 and by the CAR-specific soluble protein target fusionned to mouse FC. Cell were then washed and labeled with a secondary antibody anti-mouse FCƳ-PE followed by cells a fixation (4% PFA). Subsequent flow cytometry analysis enabled to determine the viability of CAR positive cells among singlets. Frequency of relative viability was determined by computing the ratio indicated below.

$${\rm{Relative}}\,{\rm{viability}}=({\rm{Viability}}\,{\rm{of}}\,{\rm{CAR}}\,{\rm{positive}}\,{\rm{cells}}+{\rm{treatment}}\times {\rm{100}})/({\rm{Viability}}\,{\rm{of}}\,{\rm{CAR}}\,{\rm{positive}}\,{\rm{cells}}-{\rm{treatment}})$$

### *In vivo* experiment using BRGS xenograft model

All procedures performed on animals were in accordance with regulations and established guidelines and were reviewed and approved by Pfizer’s Institutional Animal Care and Use Committee as well as by the Animal Ethical Committtee (CETEA 89, Institut Pasteur de Paris).

A total of 60 immunodeficient BRGS mice (BALB/c Rag2^tm1Fwa^ IL-2Rγ_c_^tm1Cgn^ SIRPα^NOD^, lacking T, B and NK cells,)^26,27^, were first irradiated on D1 by sub-lethal dose of Ƴ beams (4 Gy) and then adoptively transferred on D0 with MM.1S-Luc-GFP tumor cells (2 × 10^6^ cells per animal in 150 µL of PBS i.v.). Tumor cells were allowed to expand until mice randomization, performed at D17 on the basis of mice sex, age and level of tumor cell outgrowth. On the same day, mice were adoptively transferred (i.v.) with either 5 × 10^6^ viable mock-transduced T-cells, 5 × 10^6^ viable CAR positive T-cells or 5 × 10^6^ viable purified CubiCAR positive T-cells (20 mice per group, number of cells given here are actual CAR positive cell numbers). Five hours after T-cell transfer, each group received an injection (i.p.) of either 10 mg/kg human IgG isotype control or 10 mg/kg RTX (10 mice per subgroup). These injections were repeated 4 times on D18, D19, D20 and D21.

MM.1S-Luc-GFP tumor cell expansion was monitored on D21, 26 and 31 by bioluminescence imaging (BLI) using XenoLight D-luciferin (PerkinElmer), injected i.p. in animals (100 µL of 15 mg/mL solution per flank) before induction of narcosis (isoflurane). Data acquisition and analysis was performed with a Spectrum-CT apparatus (Perkin Elmer) interfaced to Living Image software (Caliper). MM.1S-Luc-GFP tumor cell, mock, CAR and CubiCAR T-cells were also monitored as a function of time by flow cytometry according to the following procedure. Half of the animals – i.e. 5 satellite mice per group – were euthanized over D24–25 and tissues of interest (blood, bone marrow and spleen) were collected. The other remaining halves were euthanized over D32–33 and processed according to the same procedure. Blood samples were harvested in presence of EDTA and blood leukocytes were purified on a Ficoll density gradient. Bone marrow cells were flushed from the bones using 25 G needles and 70 µm-filtered before use. Splenocytes were prepared by mechanical disruption of the organ followed by 70 µm-filtering and a Ficoll density gradient purification. Cell suspensions were analyzed by flow cytometry, either from a known number of cells (bone marrow, spleen) after automated counting or in presence of counting beads (blood).

A monoclonal antibody cocktail (supplementary Fig. 16a) was applied on the purified leucocytes in presence of Fc-Block reagents targeting human (1:50 diluted ‘FcR blocking reagent, human’ – Miltenyi Biotec) and murine (1:10 diluted ‘anti-mCD16/CD32 CF11 clone’ – internal source) Fc receptors. Incubations were performed in 96-well plates, in the dark and at 4 °C for 15–20 min. SA-PE-Cy7 reagent were applied separately. The cells were washed by centrifugation after staining to remove the excess of monoclonal antibody cocktail, and were re-suspended in PBS for data acquisition. All data acquisitions were performed with an LSR-II Fortessa flow cytometer interfaced with the FACS-Diva software (BD Bioscience). The analysis of the data was performed using the FlowJo-9 software (TreeStar Inc.), and statistical analysis was performed with GraphPad Prism-5 software (GraphPad Software Inc.). The gating strategy consisted in isolating the viable cells among singlets before representing the hCD45/mCD45 total plot. MM.1 S cells were extracted from the hCD45-/mCD45- population (hCD45neg/lo) and identified as GFP + /CD38 + cell population. Mock transduced, CAR and CubiCAR T-cells were extracted from hCD45 + /mCD45- population (hCD45hi) and identified as CD3 + population (hCD3+). CAR and CubiCAR T-cells were then characterized further among hCD3 + using the soluble biotinylated BCMA protein labeled with PECy7 strepavidine.

## Electronic supplementary material

Supplementary information
