# Supplementary material for: A Versatile Safeguard for Chimeric Antigen Receptor T-Cell Immunotherapies
Source: Sci Rep. 2018 Jun 12;8:8972. doi: 10.1038/s41598-018-27264-w (PMC5997667; doi:10.1038/s41598-018-27264-w)
Supplement: Supplementary file 1 — Supplementary information [file 41598_2018_27264_MOESM1_ESM.docx]

**A Versatile Safeguard for Chimeric Antigen Receptor T-Cell Immunotherapies**

**Supplementary informations**

**Julien Valton^1*^, Valerie Guyot^1^, Bijan Boldajipour^3^, Cesar Sommer^3^, Thomas Pertel^3^ Alexandre Juillerat^1^, Aymeric Duclert^2^, Barbra Johnson Sasu^3^, Philippe Duchateau^2*^ and Laurent Poirot^2^**

**^1^Cellectis Inc, 430E, 29th Street, NYC, NY 10016, USA ; ^2^Cellectis S.A., 8 rue de la Croix Jarry, 75013 Paris; ^3^Pfizer Inc/Rinat, 230 E Grand Avenue, South San Francisco, CA 94114, USA**

*Corresponding authors: julien.valton@cellectis.com and philippe.duchateau@cellectis.com


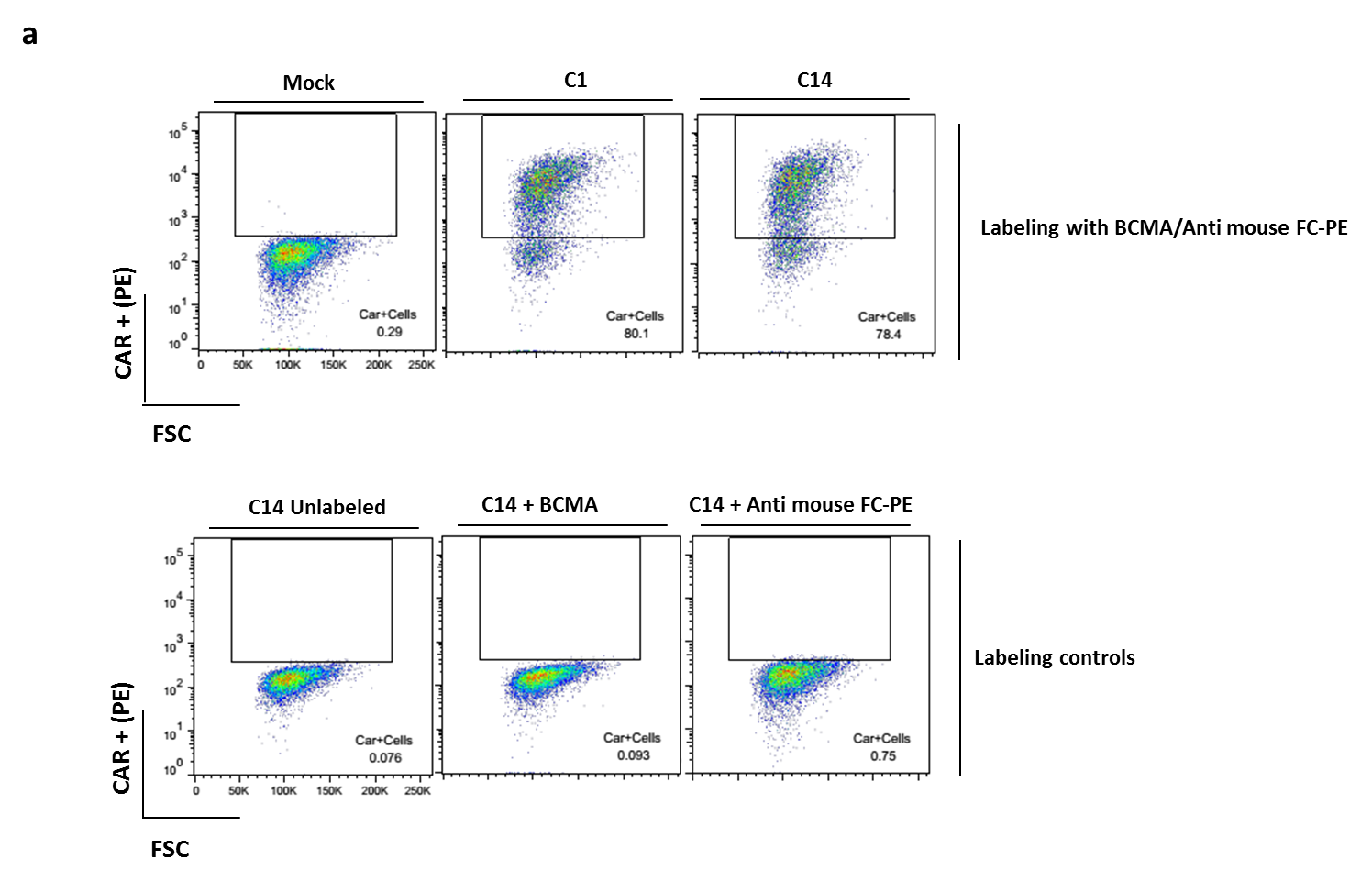


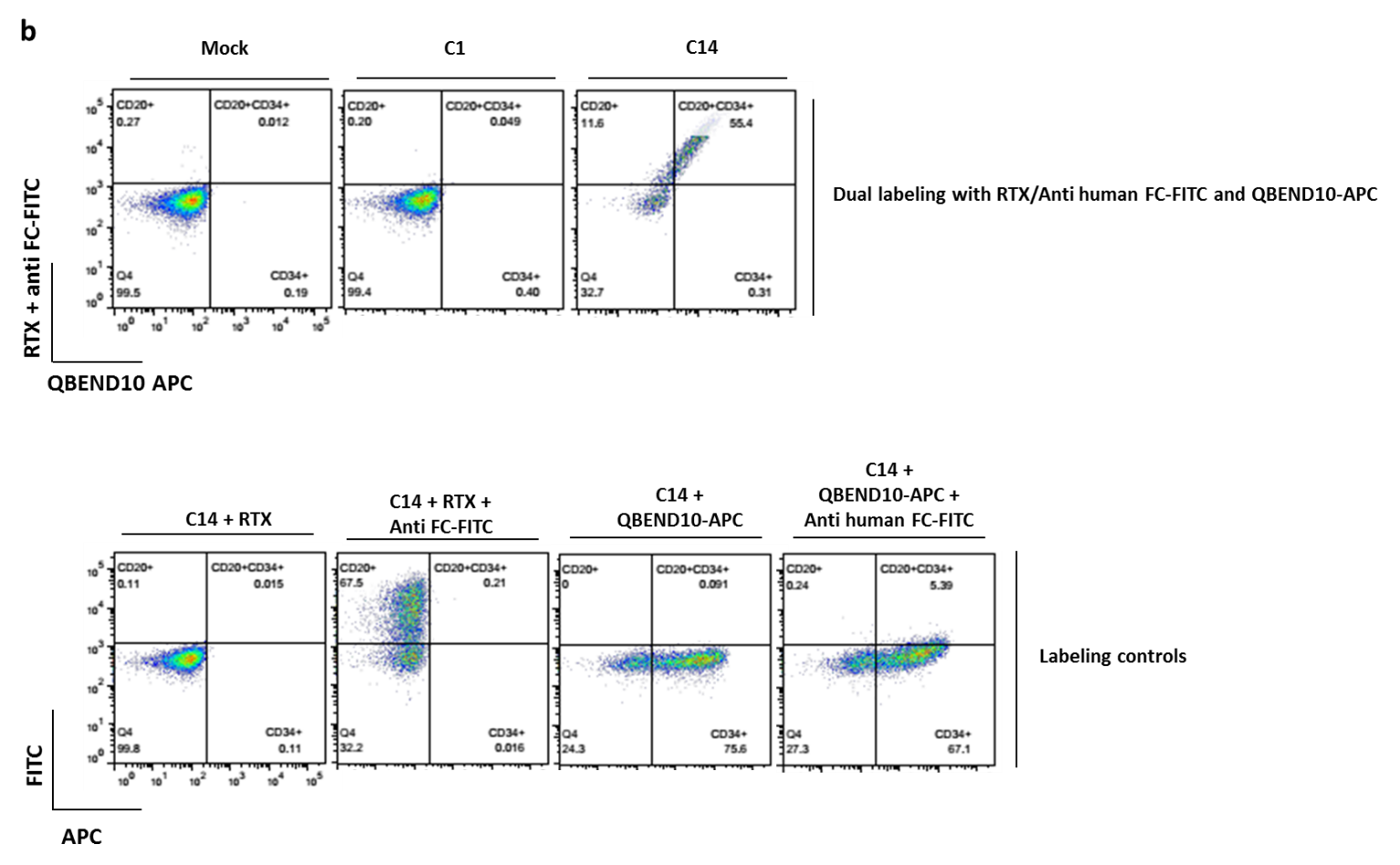


**Supplementary figure 1. Flow cytometry detection of CAR architectures transiently expressed at the surface of primary T-cells. (a)** Representative examples of mono-labeling of primary T-cells transiently expressing C1 or C14 constructs. 10^5^ T-cells were labeled with soluble BCMA-mFC protein fusion and with a PE labeled anti-FC antibody before being analyzed. **(b)** Representative examples of dual labeling of primary T-cells transiently expressing C1 or C14 constructs by RTX and QBEND10. 10^5^ T-cells were labeled with RTX and APC-labeled QBEND10 specific for CD20 and CD34 epitopes respectively. A second FITC-labeled anti-human FC was then added to detect RTX and cells were analyzed by flow cytometry. Labeling controls are illustrated on the bottom panel (gating strategy: singlets> viability>CAR+ T-cells detected by QBEND10-APC and RTX +antiFC-PE).

**
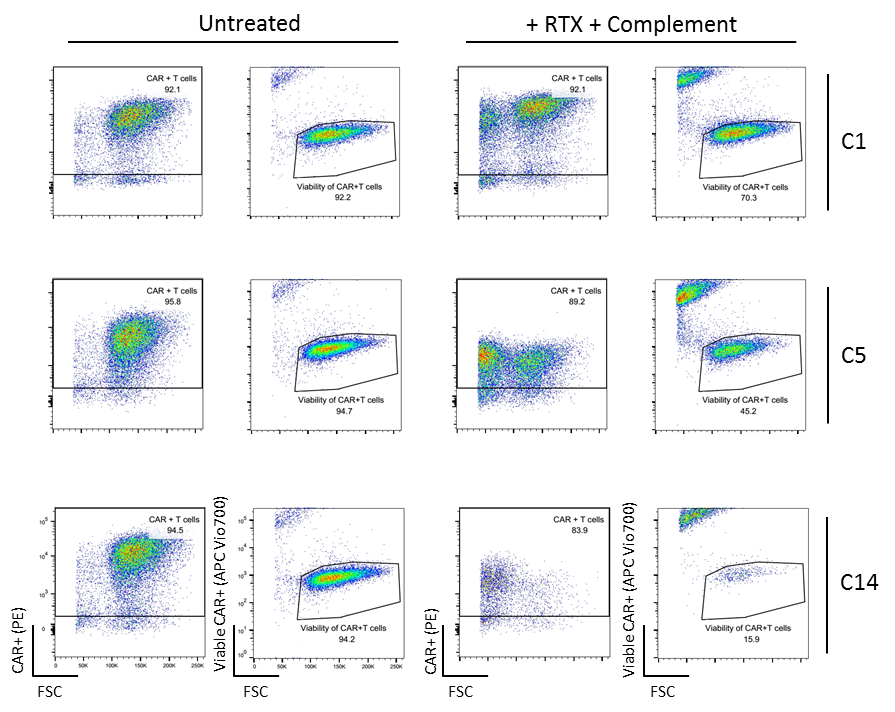
**

**Supplementary figure 2. RTX-dependent depletion of primary T-cells transiently expressing CAR architectures followed by flow cytometry.** Representative examples of flow cytometry data obtained after incubating T-cells transiently expressing C1, C5 or C14 in the presence or in the absence of RTX and complement for 150 min at 37°C. Following the treatment, T-cells were labeled by Efluor780 and analyzed by flow cytometry by looking at viable CAR+ T-cells among singlets (gating strategy: singlets>CAR+ T-cells>viability)

**Supplementary figure 3. Cytolytic activity of primary T-cells transiently expressing CAR architectures toward BCMA-expressing H929 tumor cells.** FITC-labeled BCMA^+^ target cells (H929) were co-incubated with primary T-cells transiently expressing C1-C16 CAR constructs at a E/T ratio of 10/1. After a 5 hours incubation at 37°C, cells were recovered and labeled with eFluor780 viability marker before being analysed by flow cytometry to determine their viability. Viability of BCMA^+^ target cells incubated alone or in the presence of mock transfected T-cells are also shown as negative control references. Error bars represent the standard deviation on the mean value of viability computed out of ≥2 biological replicates performed with ≥2 different donors. The significance of the differences between subgroups was assessed using a one-way ANOVA statistical test (*p<0.05, **p<0.01, ***p<0.0005, ****p<0.0001).

**
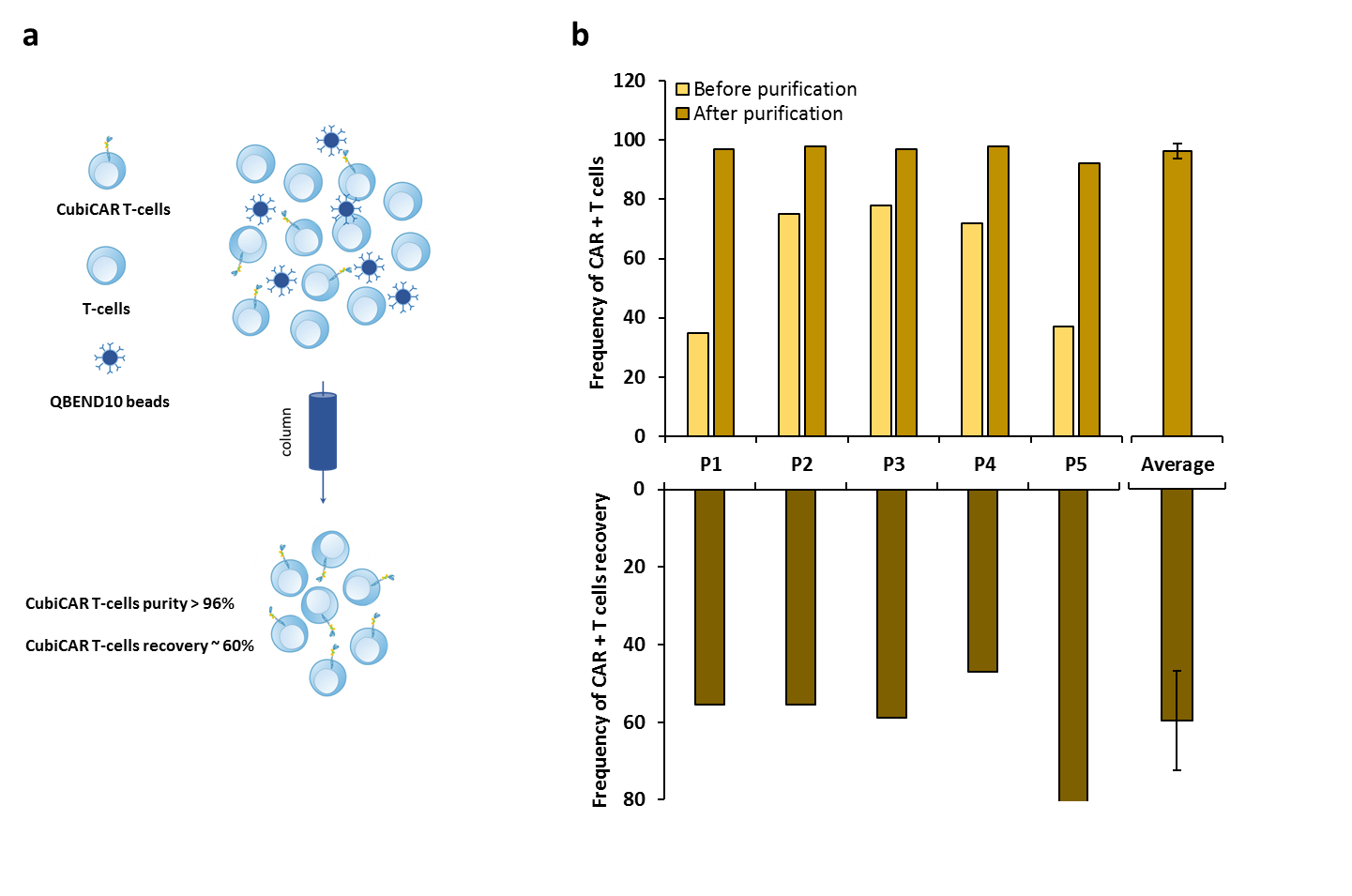
**

**Supplementary figure 4. Primary T-cells steadily expressing the CubiCAR can be efficiently purified by a GMP-compatible CD34^+^ isolation kit. (a)** Scheme of CAR+ T-cells purification process. **(b, top panel)** Frequency of CAR+ T-cells determined by flow cytometry before and after purification using the CD34^+^ isolation kit (Miltenyi). The average frequency of CAR+ T-cells obtained out of 5 independent purification experiments (P1-P5) is illustrated on the right. **(b, bottom panel)** Individual and average frequency of CAR+ T-cells recovery obtained out of 5 different purification experiments.

**
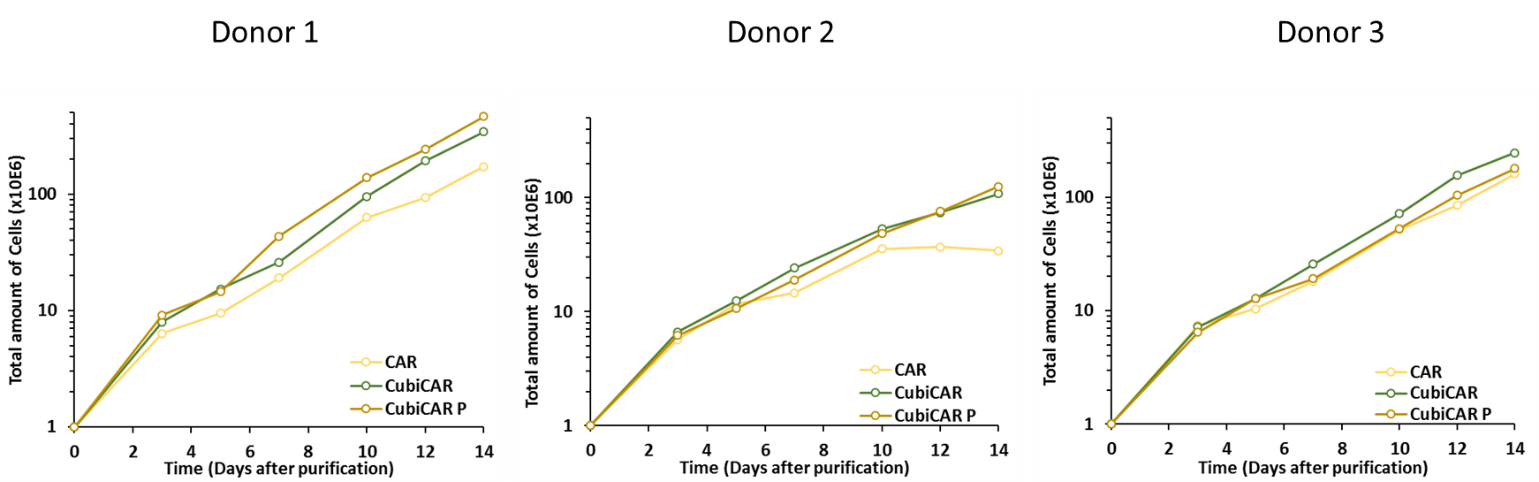
**

**Supplementary figure 5. Purified and unpurified CubiCAR T-cells show similar proliferation rate than CAR T-cells.** After purification, 10^6^ CubiCAR T-cells were grown in 12 well plates for 14 days along with CAR and unpurified CubiCAR T-cells controls. The number of viable cells counted after each passage is plotted as a function of time. Three independent experiments performed with T-cells obtained from 3 different donors are illustrated.

**Supplementary figure 6. The activation profile of CubiCAR T-cells is not influenced by their purification.** One day post purification, CubiCAR T-cells were analyzed by flow cytometry to determine the extent of CD69 activation marker expression at their cell surface. As positive control of activation, cell were incubated overnight with Dynabeads human T activator CD3/CD28. The same experiment was performed with mock transduced T-cells, CAR T-cells and unpurified CubiCAR T-cells as negative controls. Error bars represent the standard deviation on the mean value of CD69+ T-cells frequency computed out of 2 biological replicates performed with 2 different donors (gating strategy: singlets> viability>CAR+ T-cells detected by BCMA-FC recombinant protein + antiFC-PE>CD69-Vioblue). The significance of the differences between subgroups was assessed using a one-way ANOVA statistical test.


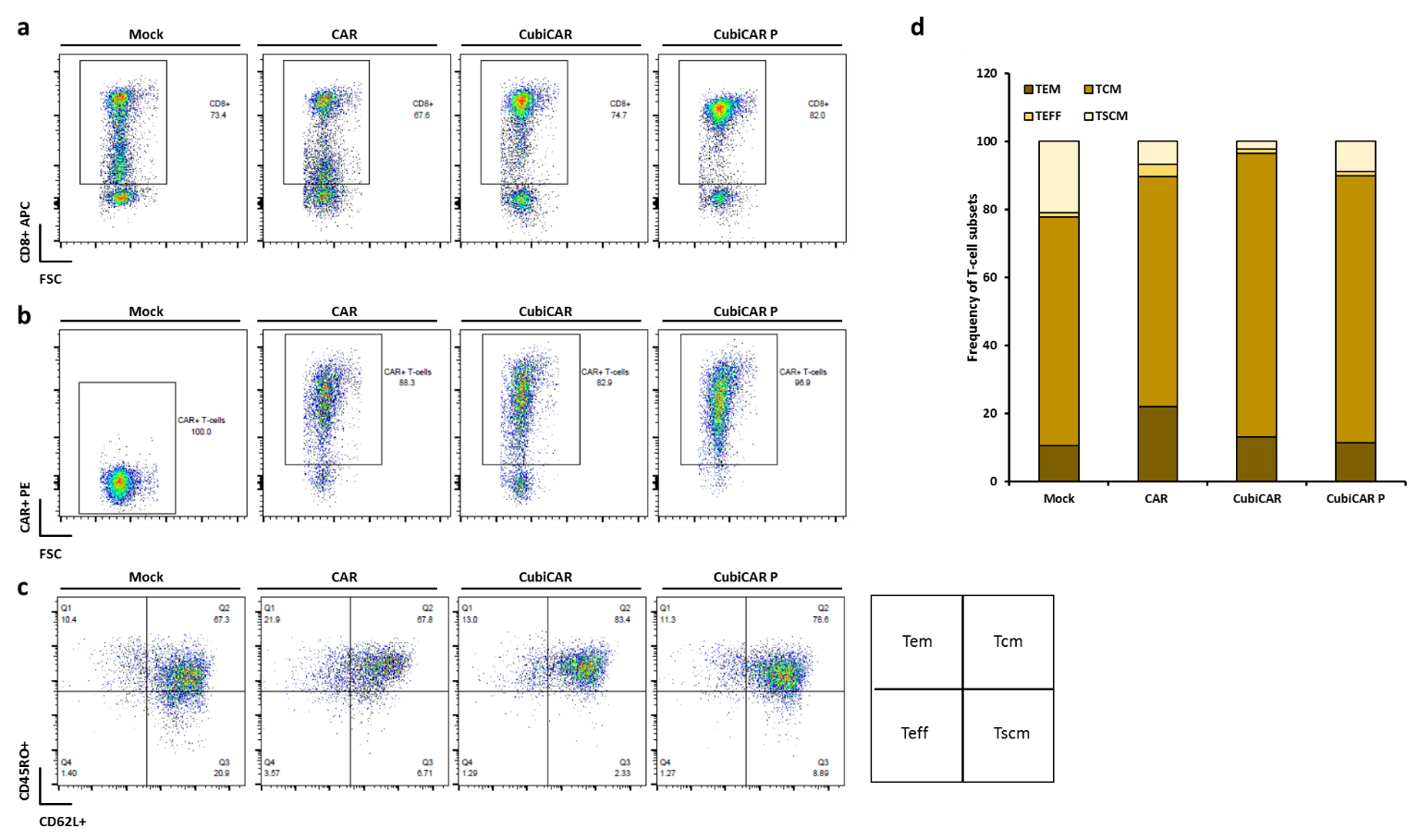


**Supplementary figure 7. Purified and unpurified CubiCAR T-cells show similar immunophenotype than CAR T-cells.** CubiCAR T-cells were grown for 14 days after purification along with unpurified CubiCAR, CAR and mock transduced T-cells. At the end of proliferation, cells were recovered and labeled by EFluor780 viability dye, anti-CD3, anti-CD8, anti-CD62L and antiCD45RO antibodies and soluble biotinylated-BCMA protein. Labeled cells were then analyzed by flow cytometry using the following gating strategy: singlets> viability >CD3+>CD8+>CAR+ T-cells> CD62L/CD45RO. **(a)** Flow cytometry plots obtained for each T-cell species. **(b)** Frequencies of stem cell memory T-cells (Tscm), central memory T-cells (Tcm), effector memory T-cells (Tem) and effector T-cells (Teff) subsets determined by flow cytometry.

| 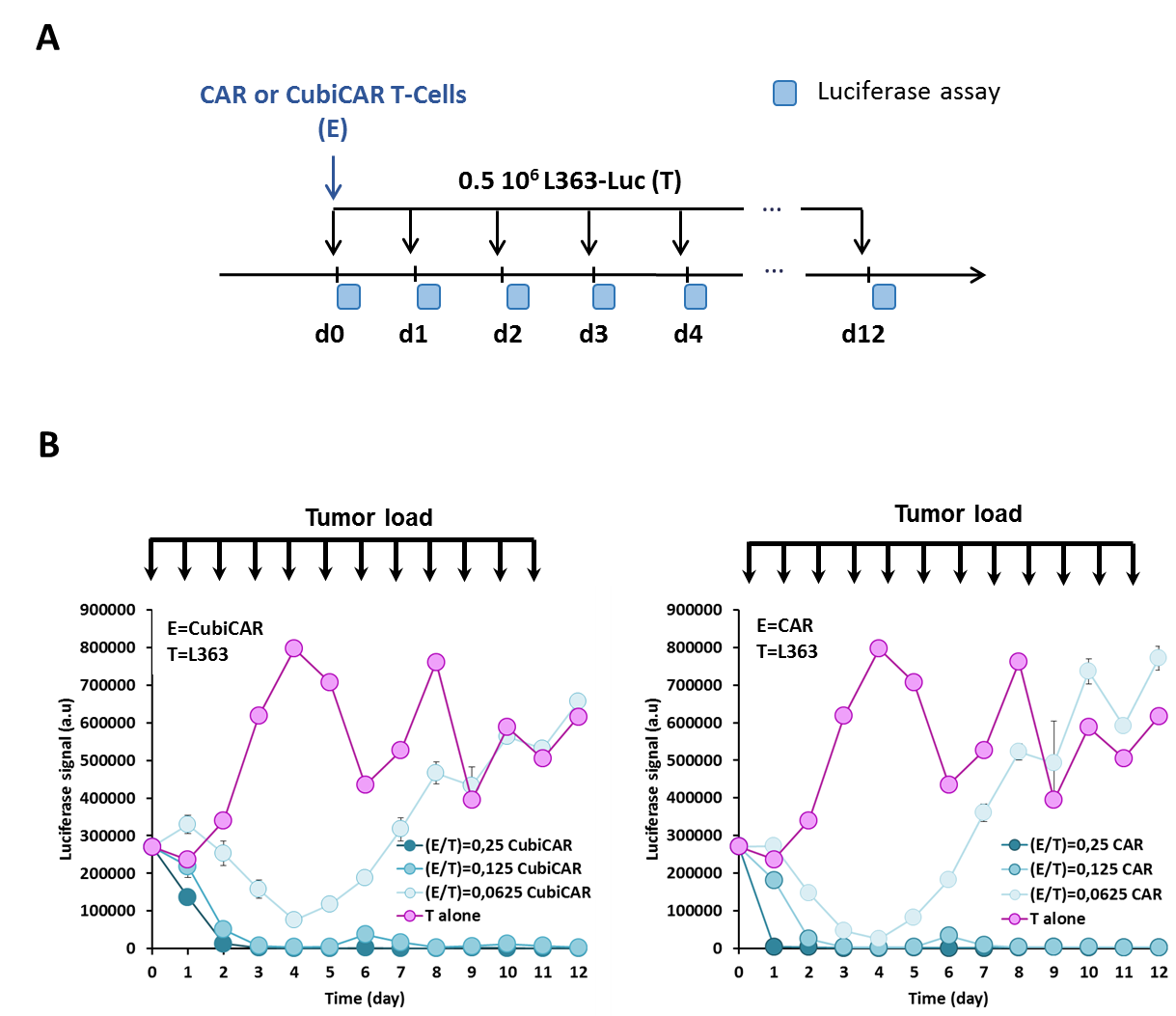 |
| --- |

**Supplemental figure 8. CAR and CubiCAR antitumor activity assessment by serial killing assays**

**(A)** scheme of the serial killing assay protocol. CAR of CubiCAR T-cells were added to a suspension of L363-Luc-GFP tumor cells (5x10^5^ total) at variable E/T ratio (E/T=0.25; 0.125; 0.062) in Xvivo-15 media supplemented by 5% AB. The mixture was incubated 24 hours before measuring the luminescence signal of the mixture. Cells were then spun down and the media supernatant was discarded and substituted with 2 mL of fresh Xvivo 5% AB containing 5x10^5^ L363-Luc-GFP cells and the resulting cell mixture was incubated for 24 hours. This protocol was repeated for 12 days. **(B)** Luminescence signals of L363-Luc cells incubated with variable amount of CubiCAR and CAR T-cells are plotted as a function of time.

| **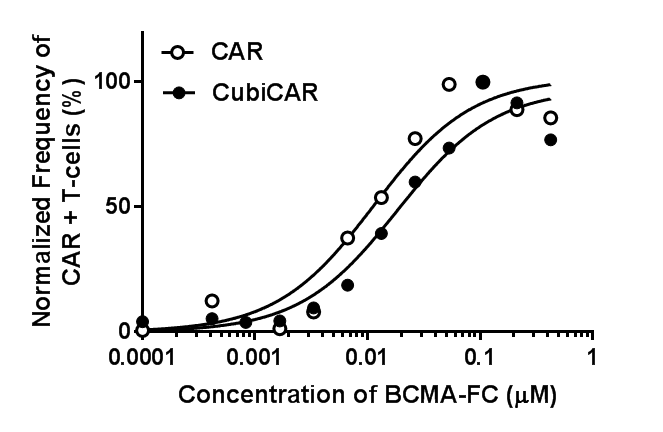** |
| --- |
| **Supplementary figure 9. Relative binding affinity of soluble BCMA-FC protein for CAR and CubiCAR T-cells.** 25000 CAR or CubiCAR T-cells were incubated for 30 minutes in the presence of increasing concentrations of soluble BCMA-FC protein (0-0.4 µM) in a final volume of 100 µL. Labeled cells were then washed and labeled by a PE-labeled anti-FC𝛄 secondary antibody. Frequency of CAR + T-cells was then determined by flow cytometry using the following gating strategy: singlets> viability > CAR+ T-cells and plotted as a function of soluble BCMA-FC concentration. Apparent BCMA EC_50_ for CAR and CubiCAR T-cell were determined to be 11.5±0.3 and 18.5±0.4 µM respectively. |

**
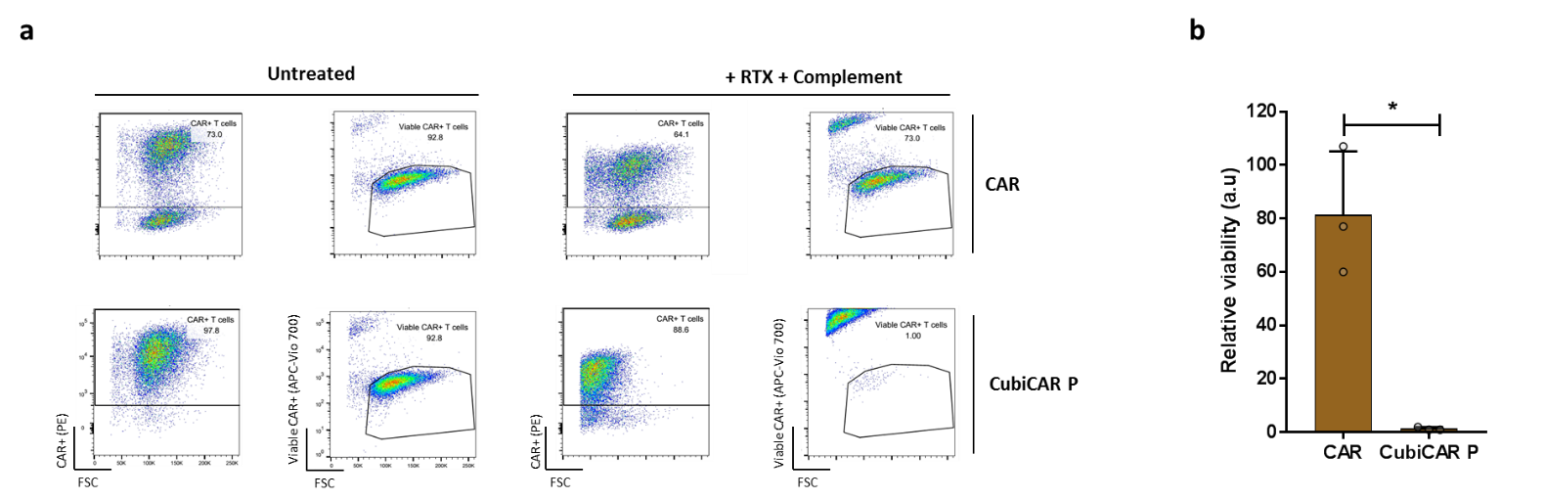
**

**Supplementary figure 10. Purified CubiCAR T-cells are efficiently depleted by RTX.**

Transduced CAR or purified CubiCAR T-cells (2x10^5^) were incubated for 150 min at 37°C alone or in the presence of 100 µg/mL RTX and complement. Cells were recovered and labeled by EFluor780 and by soluble BCMA protein fusion to mouse FC (subsequently labeled by a secondary anti-mouse FCƳ-PE antibody). Flow cytometry analysis enabled to determine the viability of CAR positive cells among singlets (gating strategy: singlets> CAR+> viability. **(a)** representative flow cytometry results. **(b)** Frequency of relative viability (see Methods section) obtained for CAR and CubiCAR T-cells. Error bars represent the standard deviation on the mean relative viability computed out of 3 biological replicates using a t-test (*p<0.05).


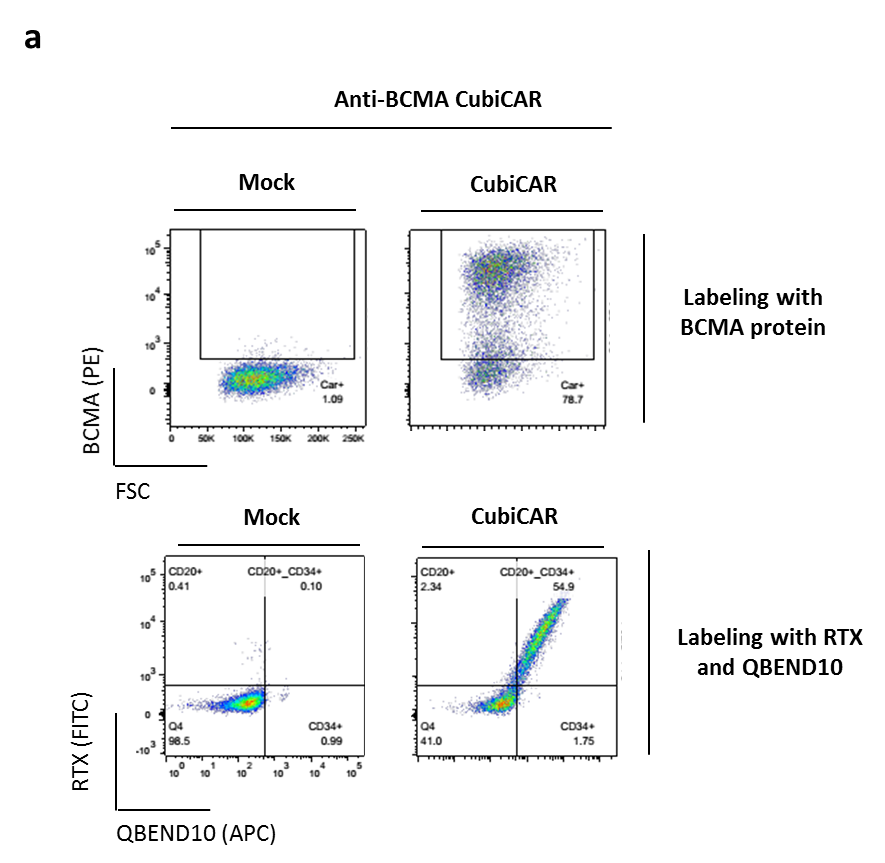


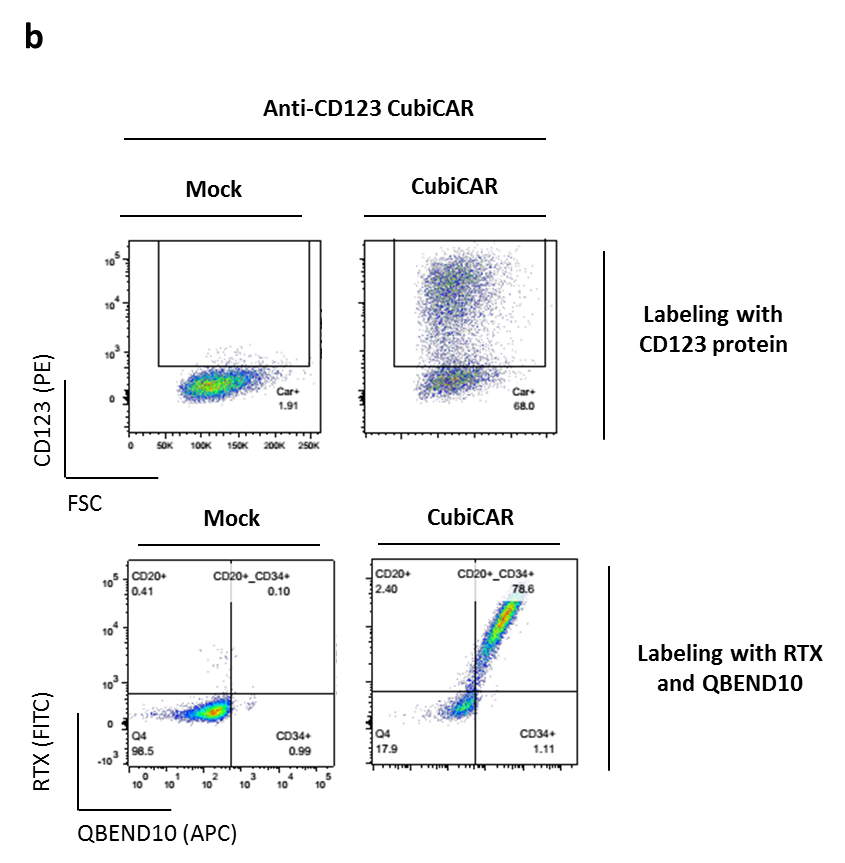


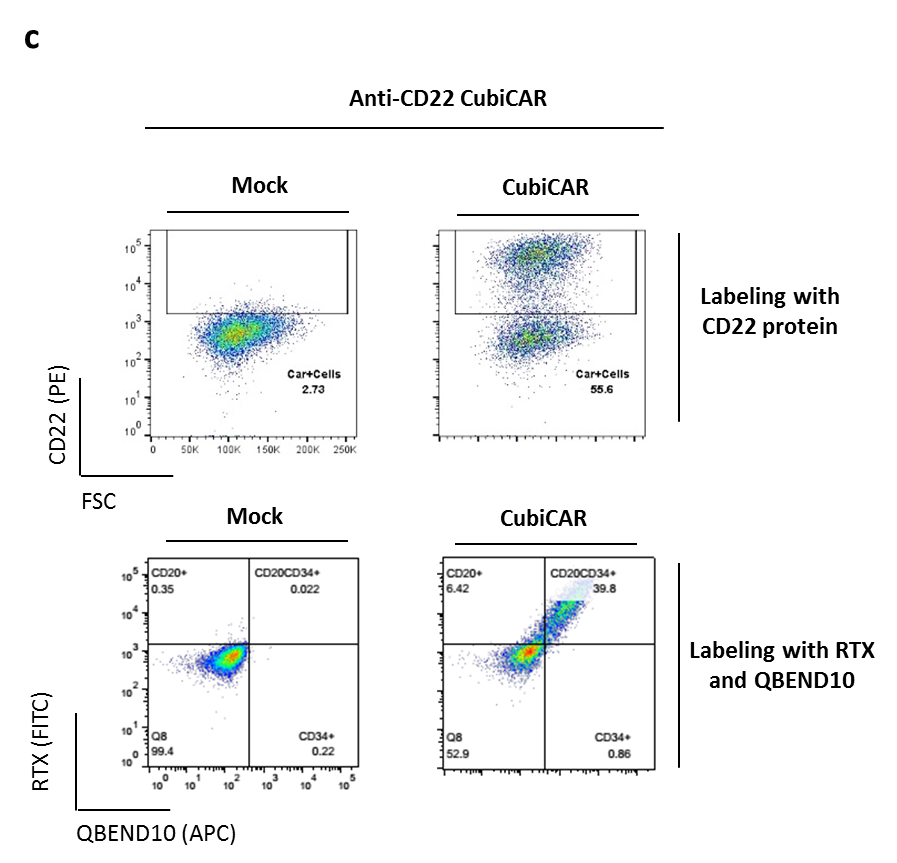


**
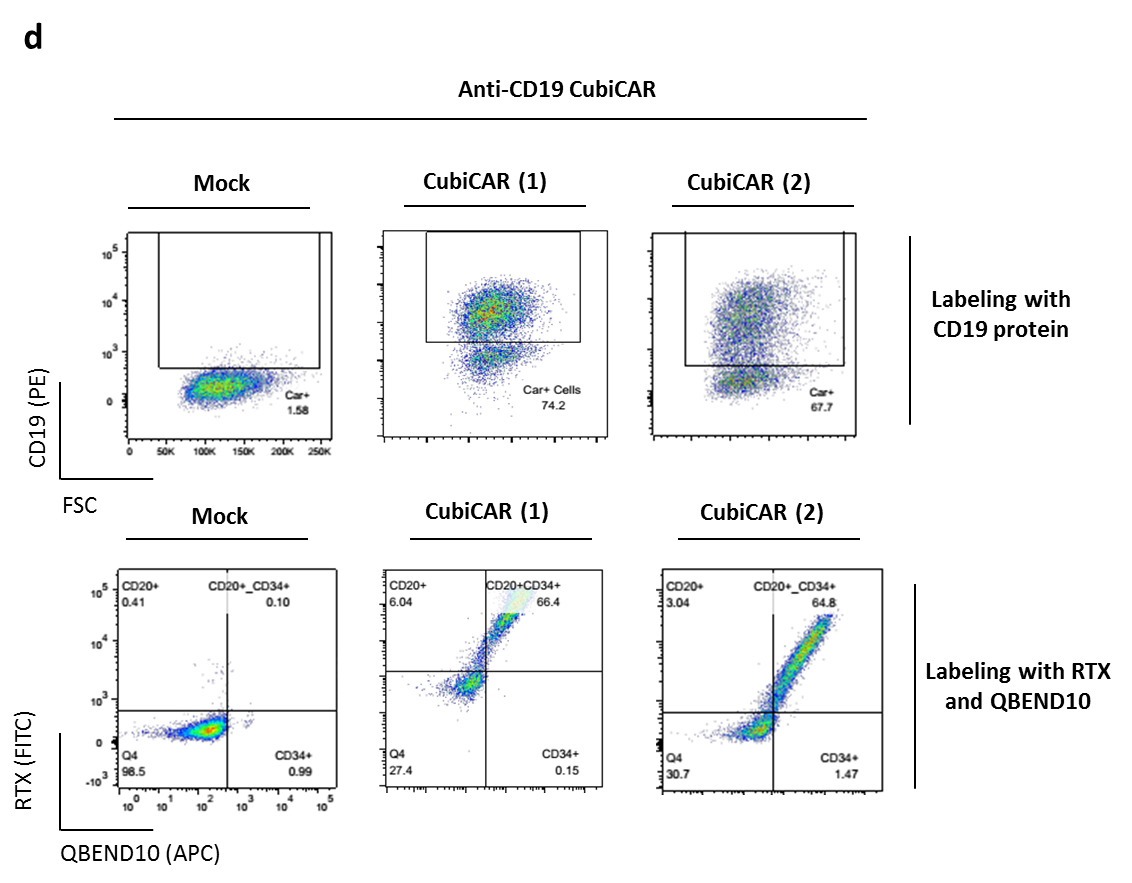
**

**Supplementary figure 11. The CubiCAR architecture can be implemented to other scFvs and detected at the surface of T-cells using QBEND10/RTX dual labeling or soluble tumor target as surface markers.** Primary T- cells transduced by lentiviral particles encoding **(a)** anti-BCMA, **(b)** anti-CD123, **(c)** anti CD22, and **(d)** 2 different anti-CD19 CubiCARs were analyzed by flow cytometry 4 days post transduction using either their respective soluble tumor target protein-FC fusion or a mixture of QBEND10-APC and RTX. Soluble tumor target protein-mFC fusion and RTX were labeled by anti-mouse FCƳ-PE and anti-human FCƳ-FITC secondary antibodies respectively. Labeled cells were analyzed using the following gating strategy: singlets > viability > soluble tumor protein+ or QBEND10+/RTX+.

**
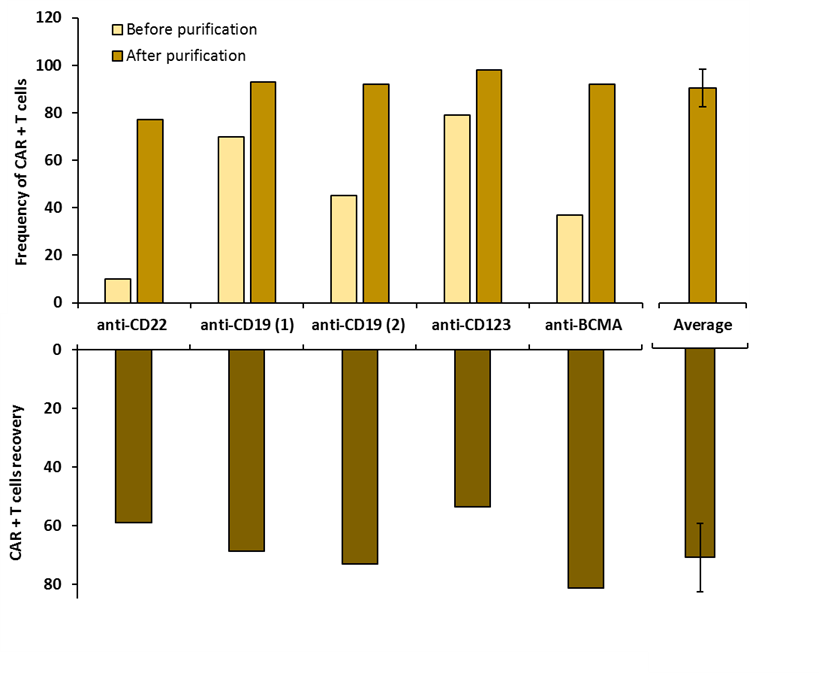
**

**Supplementary figure 12. Alternative CubiCAR T-cells can be efficiently purified by a GMP-compatible CD34^+^ isolation kit.** Frequency of alternative CubiCAR T-cells determined by flow cytometry before and after purification using the CD34^+^ isolation kit (Miltenyi). The mean frequency of CubiCAR T-cells obtained out of 5 independent purification experiments is illustrated on the right.

**
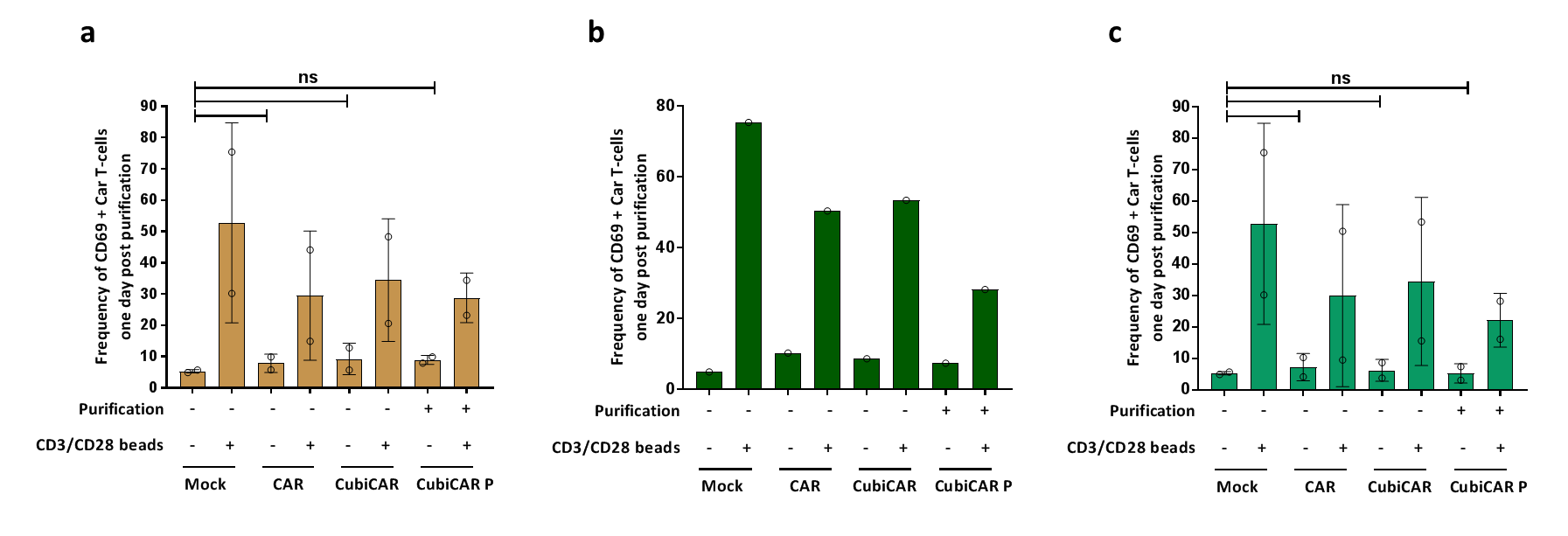
**

**Supplementary figure 13. The activation profiles of alternative CubiCAR T-cells are not influenced by their purification.** One day post purification, **(a)** anti-CD19 (2), **(b)** anti-CD22 and **(c)** anti-CD123 alternative CubiCAR T-cells were analyzed by flow cytometry to determine the extent of CD69 activation marker expression at their cell surface (gating strategy: singlets> viability>CAR+ T-cells detected by m-FC recombinant protein (CD123, CD19 or CD22) + antiFC-PE>CD69-Vioblue). As positive control of activation, cells were incubated overnight with Dynabeads human T activator CD3/CD28. The same experiment was performed with mock transduced T-cells, CAR T-cells and unpurified CubiCAR T-cells as negative controls. Error bars documented in **(a)** and **(c)** represent the standard deviation on the mean frequency of CD69+ T-cells computed out of 2 biological replicates performed with 2 different donors. Results illustrated in **(b)** were obtained from one single experiment. The significance of the differences between subgroups was assessed using a one-way ANOVA statistical test.

**
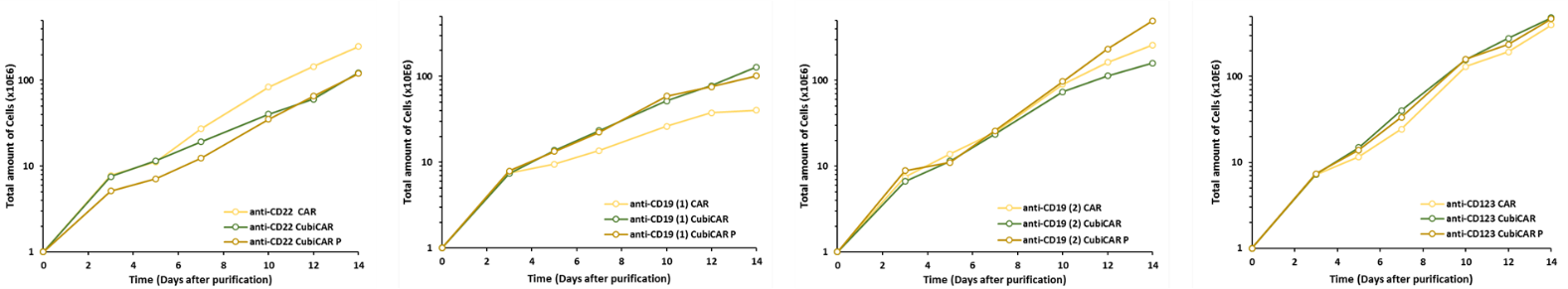
**

**Supplementary figure 14. The proliferation rates of alternative CubiCAR T-cells are not influenced by their purification.** After purification, 10^6^ of alternative CubiCAR T-cells were grown in 12 well plates for 14 days along with their CAR and unpurified CubiCAR T-cells counterparts. The number of viable cells counted after each passage is plotted as a function of time.

**Supplementary figure 15. Alternative CubiCAR T-cells can be efficiently and rapidly depleted by RTX.** Transduced CAR or purified alternative CubiCAR T-cells (2x10^5^) were incubated for 150 min at 37°C alone or in the presence of 100 µg/mL RTX and complement. Cells were recovered and labeled by EFluor780 and by their respective soluble tumor target fusionned to mFC (subsequently labeled by a secondary anti-mouse FCƳ-PE antibody). Subsequent flow cytometry analysis enabled to determine the viability of CAR positive cells among singlets. Frequency of relative viability (see Methods section) obtained for alternative CAR and CubiCAR T-cells are illustrated. Error bars represent the standard deviation on the mean relative viability computed out of ≥2 biological replicates expect for anti-CD19 (1) and anti-CD22 CubiCAR T-cells. The significance of the differences between subgroups was assessed using a one-way ANOVA statistical test (*p<0.05, **p<0.01, ***p<0.0005, ****p<0.0001)


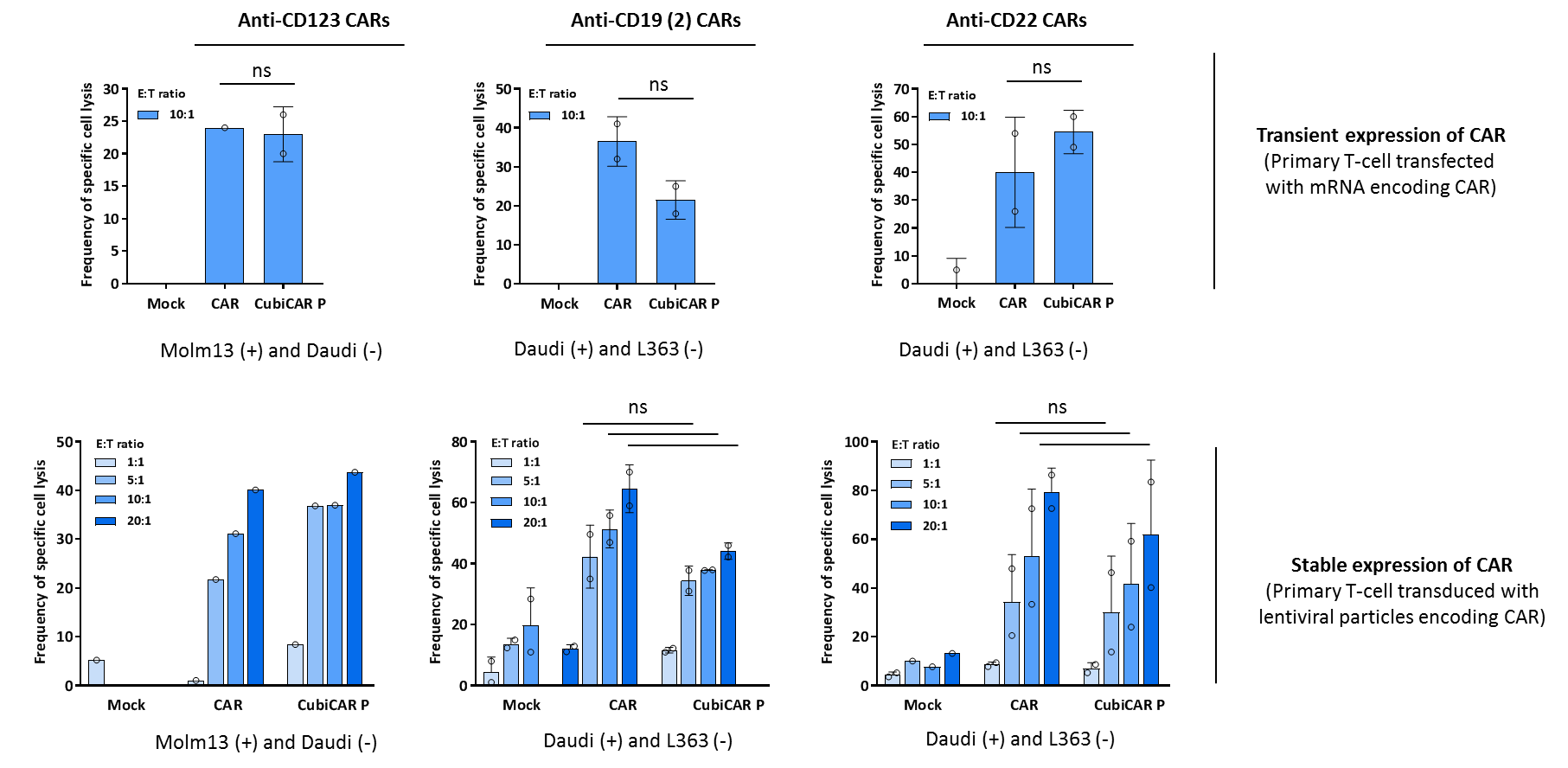


**Supplementary figure 16. Alternative CubiCAR T-cells show anti-tumor activity toward their respective specific target.** Specific cell lysis activity of primary T-cells transiently (upper panel) or steadily (bottom panel) expressing alternative CAR or CubiCAR architectures. (upper panel) FITC- and vioblue-labeled specific and non specific target cells (respectively) were co-incubated with primary T-cells transiently expressing CAR or CubiCAR constructs at a E:T ratio of 10:1 (upper panel) or of 1:1, 5:1, 10:1 and 20:1 (bottom panel). After a 5 hours incubation at 37°C, cells were recovered and labeled with eFluor780 viability marker before being analysed by flow cytometry to determine their viability. Specific cell lysis (see Methods) obtained are illustrated and error bars represent the standard deviation on the mean specific cell lysis values computed out of 2 biological replicates except for anti-CD123 CAR and anti-CD123 CubiCAR transduced T-cells that were assayed once. The significance of the differences between subgroups was assessed using a one-way ANOVA test (ns, non significant)

**
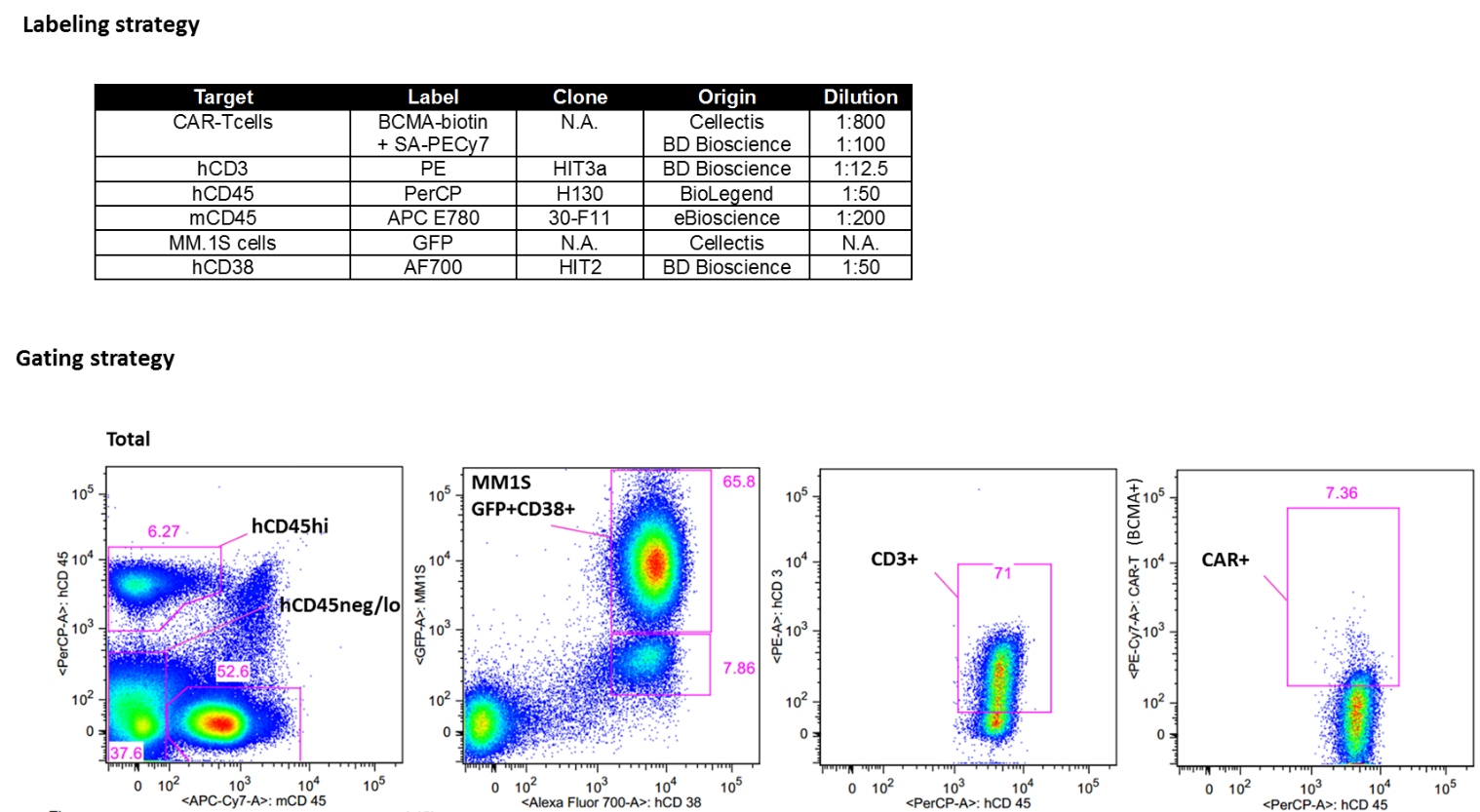
**

**
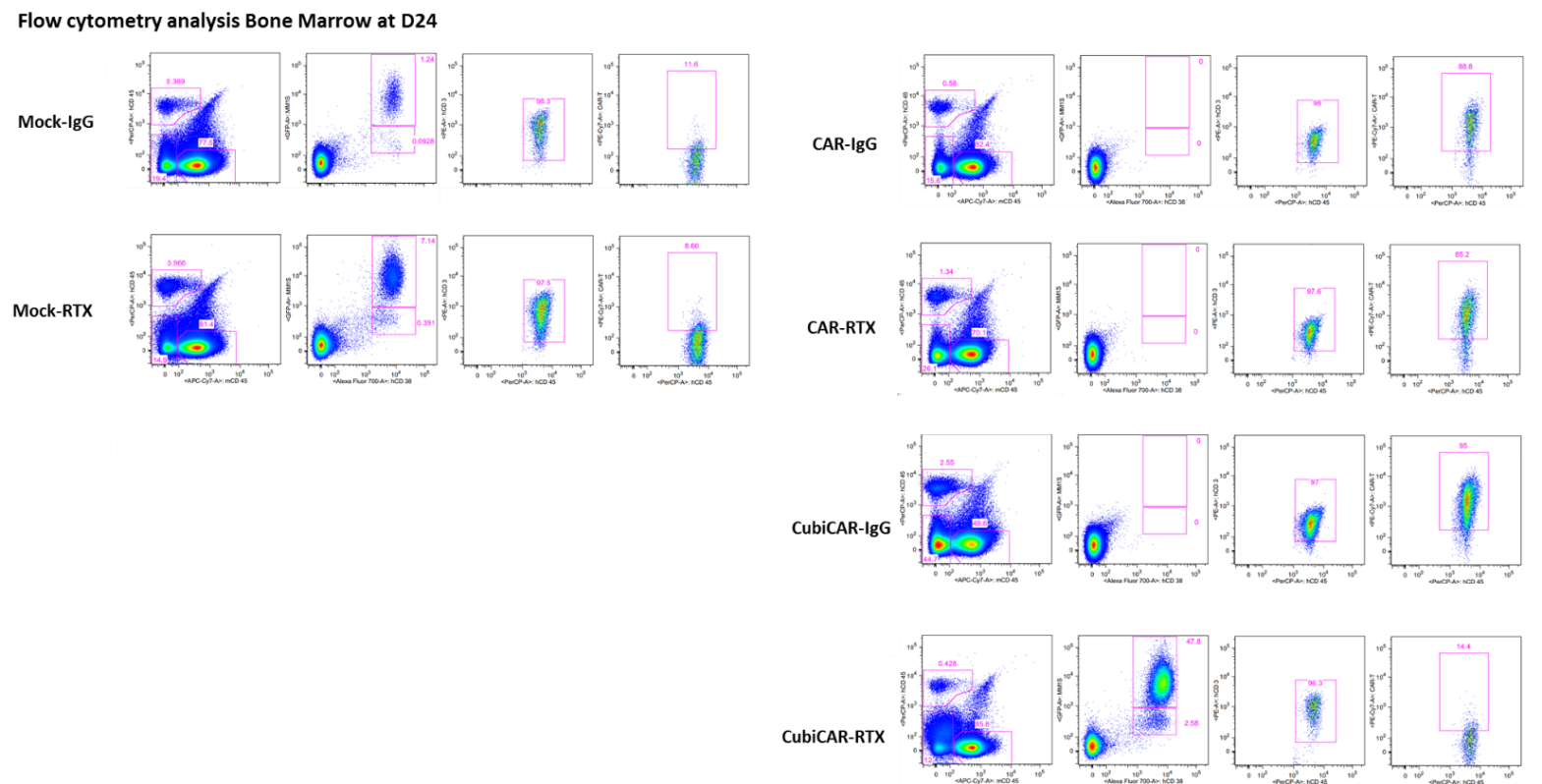
**

**Supplementary figure 18. *In vivo* characterization of CAR and purified CubiCAR T-cells activity and depletability by RTX. Upper panel,** Flow cytometry panel used to detect MM.1S tumor cells, CAR, purified CubiCAR and Mock transduced T-cells and the gating strategy employed to decipher and identify the different cellular entities from mice blood, bone marrow and spleen (BLD, BM, SPL). MM.1S-Luc-GFP cells were detected as CD38+ and GFP+ among mCD45-/hCD45- cells. CAR and CubiCAR T-cells were detected by the soluble biotinylated BCMA protein staining (BCMA+) among the hCD45+/CD3+ cells. Mock transduced T-cells (Mock) were detected among hCD45+ cells as CD3+/BCMA- cells. **Lower panel**, representative flow cytometry analysis of bone marrow obtained at D24 from the 6 different conditions including Mock transduced T-cells + IgG or RTX, CAR T-cells + IgG or RTX and purified CubiCAR T-cells + IgG or RTX.

**Supplementary Tables**

**Suplementary table I.** Characteristics of safeguard systems used in combination with CAR expression.

| **Name** | **Architecture** | **Payload (aminoacids)** | **Activating agent name/FDA compliancy** | **Allow T-cell detection/enrichement** | **Potential immunogenicity** |
| --- | --- | --- | --- | --- | --- |
| **CD20**[**^10^**](#_ENREF_10)**^,^** [**^11^**](#_ENREF_11) | 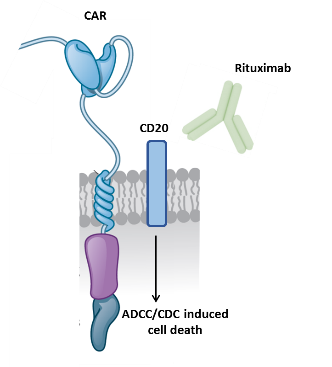 | 300 | RTX/Yes | Yes/Yes | No |
| **RQR8**[^9^](#_ENREF_9)^,^ [^22^](#_ENREF_22) | 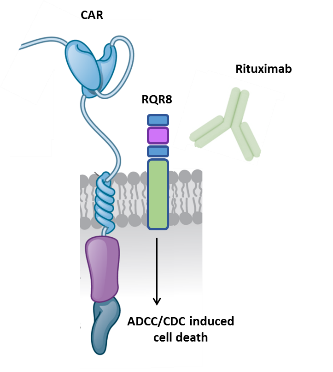 | 136 | RTX/Yes | Yes/Yes | unlikely |
| **huEGFRtr**[^7^](#_ENREF_7)^,^ [^8^](#_ENREF_8) | 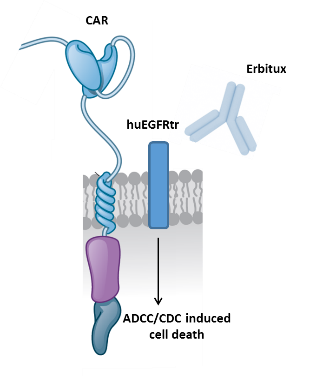 | 336 | Erbitux/Yes | Yes/Yes | No |
| **HSV-TK**[**^6^**](#_ENREF_6) | 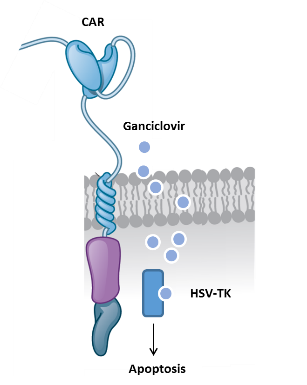 | 182 | Ganciclovir/Yes | No/No | Yes |
| **ICasp9**[**^4^**](#_ENREF_4)**^,^** [**^5^**](#_ENREF_5)**^,^** [**^30^**](#_ENREF_30) | 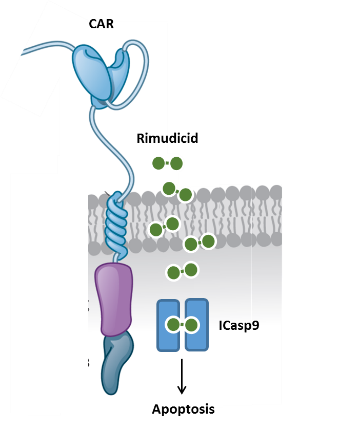 | 441 | Rimudicid/No | No/No | No |

**Suplementary table II**. Names, subgroup names and protein sequences of anti-BCMA CAR constructs assembled by golden gate cloning and tested in this study. Amino acid sequences of inter V1/V2 conventional GS linker, engineered GS linkers, CD20 mimotopes and CD34 epitopes are indicated respectively in blue, green, purple and dark purple. 0m, 1m, 2cm, 2sm, 3cm and 3sm mean respectively that CAR construct contain no mimotope, 1 mimotope, 2 consecutive mimotopes (separated by short SGGGGS linker), 2 separated mimotopes (separated by linker longer than SGGGGS linker), 3 consecutive mimotopes (separated by short SGGGGS linker) and 3 separated mimotope (separated by linker longer than SGGGGS linker). For the sake of clarity, the names of signal sequence (SSeq), V1 and V2 (constituting the ScFv), CD8 hinge, CD8 transmembrane (TM), 41BB and CD3zeta (CD3z) domains are indicated and color coded in grey, yellow and azul. The additional aminoacids generated by the restriction sites used to assembled construct by golden gate cloning (AP and SDP) are also indicated.

| **Construct name** | **Construct Subgroup** | **Protein sequence of additional amino acids inserted in CAR constructs** |
| --- | --- | --- |
| C1 | 0m | SSeq-V1-SSGGGGSGGGGSGGGGS-V2-AP-CD8HINGE/TM-41BB-CD3Z |
| C2 | 1m | SSeq-V1-SSGGSCPYSNPSLCSGGS-V2-SDP-CD8HINGE/TM-41BB-CD3Z |
| C3 | 1m | SSeq-V1-SSGGGGGSCPYSNPSLCSGGGGGS-V2-SDP-CD8 HINGE/TM-41BB-CD3Z |
| C4 | 2cm | SSeq-V1-SSGGGGSGGGGSGGGGS-V2-SDP-GSGGGGSCPYSNPSLCSGGGGSCPYSNPSLC-AP-CD8HINGE/TM-41BB-CD3Z |
| C5 | 2cm | SSeq-V1-SSGGGGSGGGGSGGGGS-V2-SDP-GSGGGGSCPYSNPSLCSGGGGSCPYSNPSLCSGGGGS-AP-CD8HINGE/TM-41BB-CD3Z |
| C6 | 2cm | SSeq-GGGGSCPYSNPSLCSGGGGSCPYSNPSLCSGGGGS-V1-SSGGGGSGGGGSGGGGS-V2-SDP-CD8HINGE/TM-41BB-CD3Z |
| C7 | 3cm | SSeq-V1-SSGGGGSGGGGSGGGGS-V2-SDP-GSGGGGSCPYSNPSLCSGGGGSCPYSNPSLCSGGGGSCPYSNPSLC-AP-CD8HINGE/TM-41BB-CD3Z |
| C8 | 2sm | SSeq-V1-SSGGGGSGGGGSGGGGS-V2-SDP-GSGGGGSCPYSNPSLCSGGGGSELPTQGTFSNVSTNVSPAKPTTTACPYSNPSLC-AP-CD8HINGE/TM-41BB-CD3Z |
| C9 | 2sm | SSeq-GGGGSCPYSNPSLCSGGGGSGGGGS-V1-SSGGSCPYSNPSLCSGGS-V2-SDP-CD8HINGE/TM-41BB-CD3Z |
| C10 | 2sm | SSeq-GGGGSCPYSNPSLCSGGGGSGGGGS-V1-SSGGGGGSCPYSNPSLCSGGGGGS-V2-SDP-CD8HINGE/TM-41BB-CD3Z |
| C11 | 2sm | SSeq-V1-SSGGSCPYSNPSLCSGGS-V2-SDP-GSGGGGSCPYSNPSLCSGGGGSAP-CD8HINGE/TM-41BB-CD3Z |
| C12 | 2sm | SSeq-V1-SSGGGGGSCPYSNPSLCSGGGGGS-V2-SDP-GSGGGGSCPYSNPSLCSGGGGS-AP-CD8HINGE/TM-41BB-CD3Z |
| C13 | 2sm | SSeq-GGGGSCPYSNPSLCSGGGGSGGGGS-V1-SSGGGGSGGGGSGGGGS-V2-SDPGSGGGGSCPYSNPSLCSGGGGS-AP-CD8HINGE/TM-41BB-CD3Z |
| C14 | 3sm | SSeq-GGGGSCPYSNPSLCSGGGGSGGGGS-V1-SSGGGGSGGGGSGGGGS-V2-SDP-GSGGGGSCPYSNPSLCSGGGGSELPTQGTFSNVSTNVSPAKPTTTACPYSNPSLC-AP-CD8HINGE/TM-41BB-CD3Z |
| C15 | 3sm | SSeq-GGGGSCPYSNPSLCSGGGGSGGGGS-V1-SSGGSCPYSNPSLCSGGS-V2-SDP-GSGGGGSCPYSNPSLCSGGGGS-AP-CD8HINGE/TM-41BB-CD3Z |
| C16 | 3sm | SSeq-GGGGSCPYSNPSLCSGGGGSGGGGS-V1-SSGGGGGSCPYSNPSLCSGGGGGS-V2-SDP-GSGGGGSCPYSNPSLCSGGGGS-AP-CD8HINGE/TM-41BB-CD3Z |
